# Excisionase in Pf filamentous prophage controls lysis‐lysogeny decision‐making in *Pseudomonas aeruginosa*

**DOI:** 10.1111/mmi.14170

**Published:** 2018-12-12

**Authors:** Yangmei Li, Xiaoxiao Liu, Kaihao Tang, Pengxia Wang, Zhenshun Zeng, Yunxue Guo, Xiaoxue Wang

**Affiliations:** ^1^ Key Laboratory of Tropical Marine Bio‐resources and Ecology, Guangdong Key Laboratory of Marine Materia Medica, RNAM Center for Marine Microbiology, South China Sea Institute of Oceanology Chinese Academy of Sciences Guangzhou 510301 PR China; ^2^ University of Chinese Academy of Sciences Beijing 100049 China

## Abstract

Pf filamentous prophages are prevalent among clinical and environmental *Pseudomonas* *aeruginosa *isolates. Pf4 and Pf5 prophages are integrated into the host genomes of PAO1 and PA14, respectively, and play an important role in biofilm development. However, the genetic factors that directly control the lysis‐lysogeny switch in Pf prophages remain unclear. Here, we identified and characterized the excisionase genes in Pf4 and Pf5 (named *xisF4* and *xisF5*, respectively). XisF4 and XisF5 represent two major subfamilies of functional excisionases and are commonly found in Pf prophages. While both of them can significantly promote prophage excision, only XisF5 is essential for Pf5 excision. XisF4 activates Pf4 phage replication by upregulating the phage initiator gene (*PA0727*). In addition, *xisF4* and the neighboring phage repressor *c* gene *pf4r* are transcribed divergently and their 5′‐untranslated regions overlap. XisF4 and Pf4r not only auto‐activate their own expression but also repress each other. Furthermore, two H‐NS family proteins, MvaT and MvaU, coordinately repress Pf4 production by directly repressing *xisF4*. Collectively, we reveal that Pf prophage excisionases cooperate in controlling lysogeny and phage production.

## Graphical abstract

A proposed model of the lysis‐lysogeny switch of Pf4 in PAO1. Integration of Pf4 in host genome is maintained by MvaT/MvaU by binding to the excisionase gene *xisF4*. Inactivation of MvaT/MvaU upregulates *xisF4* and subsequently, XisF4 activates the replication initiator gene *PA0727*, leading to Pf4 phage replication and release.

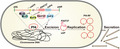

## Introduction

Filamentous phages, among the simplest biological entities known, were discovered over a half‐century ago. Profoundly different from tailed double‐stranded DNA (dsDNA) phages, filamentous phages feature long, thin filaments and a small, circular single‐stranded DNA (ssDNA) genome (Mai‐Prochnow *et al.*, 2015).The Ff filamentous phages which infect conjugative *Escherichia coli* strains, including f1, fd and M13, were isolated from sewage systems in the early 1960s (Loeb, 1960; Hofschneider and Preuss, 1963; Marvin and Hoffmann‐Berling, 1963). The *Pseudomonas aeruginosa* phage Pf1 was described in 1966 and is twice the size of Ff phages (Takeya and Amako, 1966). Subsequently, these phages have been found in a variety of Gram‐negative bacteria and occasionally in Gram‐positive bacteria in diverse habitats (Mai‐Prochnow *et al.*, 2015). Rather than kill host bacteria, filamentous phages generally become lysogens, with the phage genome either being integrated into the bacterial chromosome or remaining extrachromosomal as episomes (Mai‐Prochnow *et al.*, 2015). Increasing evidence demonstrates that they can strongly affect host physiology and virulence expression as lysogens. For example, the filamentous phage CTXφ in *Vibrio cholera* carries genes encoding the cholera toxin and plays a critical role in the conversion of nontoxigenic strains into pathogens (Waldor and Mekalanos, 1996; Davis *et al.*, 2002). Another filamentous phage, VPIφ in *V. cholera*, encodes the structural gene for a toxin‐co‐regulated pilus, which is not only the receptor for CTXφ but also a colonization factor (Li *et al.*, 2003). The filamentous phage φRSM3 in *Ralstonia solanacearum* encodes a transcriptional regulator that represses the expression of the host virulence genes (Addy *et al.*, 2012).

Pf1‐like filamentous phages are prevalent among clinical and environmental *P. aeruginosa* isolates, and more than half of the 241 clinical isolates were found to harbor at least one Pf1‐like genetic element (Knezevic *et al.*, 2015). The Pf prophage Pf1 infects *P. aeruginosa *strain K (PAK) without integrating into the host genome and replicates exclusively as an episome. In contrast, Pf4 and Pf5 are integrated into the host genomes of PAO1 and PA14, respectively, and maintained as prophages (Mooij *et al.*, 2007). During *P. aeruginosa* biofilm development, Pf1‐like genes are among the most strongly induced genes (Whiteley *et al.*, 2001). Pf4 in PAO1 plays an essential role in biofilm development and the structural integrity of the biofilm (Rice *et al.*, 2009). During biofilm formation, Pf4 can develop into mature virus particles and then can convert into a superinfective form that is correlated with cell death and the appearance of small‐colony variants (SCVs) (Webb *et al.*, 2004). Pf4 also contributes to the virulence of PAO1, as shown by the increased survival of the strain without Pf4 using a mice infection model (Rice *et al.*, 2009). More specifically, Pf4 can promote bacterial adhesion to mucin, alter progression of the inflammatory response, and contribute to noninvasive infection in a murine pneumonia model (Secor *et al.*, 2017). Furthermore, the production of Pf4 phage during PAO1 biofilm development is found to be associated with the formation of highly ordered liquid crystals, thus promoting the pathogenic features of biofilms (Secor *et al.*, 2015). In addition, inactivation of Pf5 genes increases biofilm formation in PA14 (Lee *et al.*, 2018). It has become clear that a mutualistic relationship has developed between *P. aeruginosa* and the Pf prophages.

Phage integration is a crucial step for lysogeny, while prophage excision is a critical step for phage production (Feiner *et al.*, 2015). For tailed dsDNA phages, prophage excision requires integrase and additional recombinase activity normally conferred by a phage‐encoded recombination directionality factor (RDF) or excisionase (Bertani and Bertani, 1971; Gottesman, 1974; Couturier, 1976). Filamentous phages have smaller genomes than tailed dsDNA phages, and many of them do not encode their own recombinases. In some cases, host‐encoded recombinases are needed for prophage excision. For example, excision of filamentous prophages CTXφ and VGJφ in *Vibrio cholera* and XacF1 in *Xanthomonas* *axonopodis* are mediated by the host‐encoded recombinases XerC/D (Huber and Waldor, 2002; Das *et al.*, 2011; Ahmad *et al.*, 2014). For Pf prophages, nonintegrated Pf1 prophage carries a truncated integrase but Pf4 and Pf5 prophages both carry intact integrases. However, it remains unknown whether Pf prophages encode their own excisionases or use the host recombinase to mediate excision.

Prophages can maintain two distinct life forms: a lytic cycle and a lysogenic cycle. Although in most cases, the lysogenic state is relatively stable, alterations in host cell physiology may lead to the lytic cycle which initiates phage production and/or host cell lysis (Ofir and Sorek, 2018). The best‐studied lysis‐lysogeny conversion is between lambda prophage and its *E. coli* host, and this conversion is regulated by host‐encoded proteins RecA and LexA under the SOS response (Little and Mount, 1982). In recent years, several studies showed that several prophages induced during biofilm development are specifically regulated by the host‐encoded histone‐like nucleoid structuring (H‐NS) family proteins (Wang *et al.*, 2009; Hong *et al.*, 2010; Liu *et al.*, 2015; Zeng *et al.*, 2016). H‐NS family proteins are shown to bind to intrinsically curved DNA to regulate the expression and excision of mobile genetic elements (Dorman, 2007; Singh and Grainger, 2013). For example, in *Salmonella enterica*, H‐NS binds to AT‐rich sequences and silences the *Salmonella* pathogenicity islands (Navarre *et al.*, 2006). In *E. coli*, H‐NS binds to the promoter region of the *tra *operon of the conjugative F plasmid, which encodes components of the transfer apparatus, resulting in the repression of plasmid transfer (Will and Frost, 2006). We found that CP4‐57 and rac prophages in *E. coli* K‐12 are excised during *E. coli* biofilm development and that these two prophages are not regulated by the SOS response (Wang *et al.*, 2010). Indeed, excision of the prophage rac is controlled by H‐NS through its binding to the rac excisionase gene *xisR *(Hong *et al.*, 2010; Liu *et al.*, 2015), while excision of the P4‐family prophage CP4‐57 in *E. coli* is controlled by another DNA binding protein, Hha (Wang *et al.*, 2009), a protein known to interact with H‐NS (Nieto *et al.*, 2002) and recently shown to behave as a toxin with antitoxin TomB (Marimon *et al.*, 2016). We recently reported that the host‐encoded H‐NS represses CP4So prophage excision in* Shewanella oneidensis* by binding to the excisionase gene *alpA *at warm temperatures, while the de‐repression of *alpA* by H‐NS induces prophage excision to increase host fitness at cold temperatures (Zeng *et al.*, 2016). In PAO1, two H‐NS family proteins, MvaT and MvaU, function coordinately as xenogeneic silencers and control the activation of Pf4 (Castang *et al.*, 2008; Li *et al.*, 2009). A previous ChIP‐chip assay also suggested that MvaT and MvaU bind to the region upstream of the Pf4 structural genes (Castang and Dove, 2012). However, the underlying mechanism of action of the H‐NS family proteins on Pf prophages remains elusive.

In this study, we identified a new ORF (*xisF4*) in the prophage Pf4 that encodes a functional excisionase. This is the first report that demonstrates that filamentous prophages carry their own excisionases. Excision of the prophage Pf4 increased 10^4^‐fold upon overexpression of *xisF4*, and deletion of *xisF4* abolished the ability of Pf4 to excise from the host chromosome. XisF4 also activates the replication of phage production by upregulating the recently reported replication initiator gene (*PA0727*). In addition, MvaT and MvaU coordinately repress Pf4 production by directly repressing the promoter activity of *xisF4*. Similarly, the excisionase XisF5 in the prophage Pf5 also induces excision of Pf5 in PA14. Taken together, we demonstrate that Pf prophages can encode their own excisionase and that host factors control Pf excision and production through the regulation of this excisionase gene.

## Results

### Identification of excisionase genes in the prophages Pf4 and Pf5

**Figure 1:**
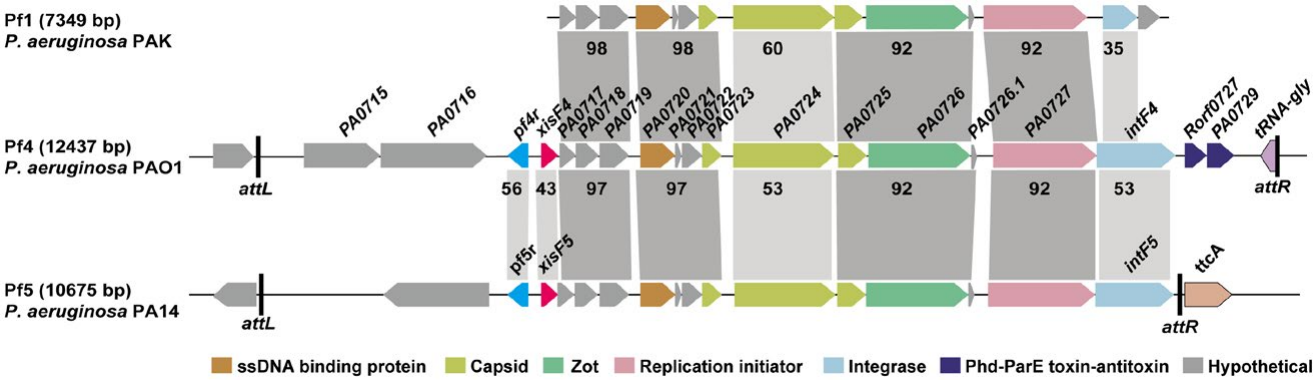
Sequence alignment of prophage genes in Pf1, Pf4 and Pf5. Names of the Pf4 genes in PAO1 are shown to scale. The reannotated genes *xisF4* and *pf4r* in PAO1 and *xisF5* and *pf5r *in PA14 are also shown here. *attL* and *attR* represent the left and right attachment sites, respectively. Numbers in the dark gray shaded regions represent the percentage of DNA sequence identity between genes (> 90%), and the numbers in the light gray shaded regions represent the percentage of amino acid sequence identity between proteins (40~60%).

Unlike Pf1 in strain PAK, Pf4 and Pf5 integrate as prophages in the genomes of PAO1 and PA14, respectively. In PAO1, Pf4 is integrated between *PA0714* (encoding a hypothetical protein) and *PA0729.1* (encoding tRNA‐gly) (Webb *et al.*, 2004). In PA14, Pf5 is integrated into a different site, between *PA14_49040* and *PA14_48870* (Mooij *et al.*, 2007). Comparative genomic analysis shows that a majority of the genes encoding structural proteins of the filamentous phages (*PA0717*‐*PA0726*) share higher sequence identity (> 90%) than the rest of the regions, except for *PA0724* which encodes the minor coat protein (~50%) (Fig. 1). Downstream of the region coding for the structural genes is *PA0727*, which was recently found to be responsible for phage replication (Martínez and Campos‐Gómez, 2016). Moreover, PA0727 also shares high sequence identity among Pf1, Pf4 and Pf5, and it encodes the phage replication initiator protein in PAO1 (Fig. 1). Downstream of *PA0727* is *PA0728* (renamed as *intF4*) which encodes a putative integrase; however, a truncated integrase gene (integrase in Pf1 is 100 aa and the integrase in Pf4 is 328 aa) is present in the phage Pf1.

We analyzed the genomic region between *PA0716* and *PA0717*. Although this region is absent in the phage Pf1, moderate sequence identity was found in a region of ~600 bp next to *PA0717* in Pf4 and Pf5 prophages. Previous analysis by the Kjelleberg group suggested that this region contained an ORF encoding putative regulatory proteins of 88 aa with 42% sequence identity with the repressor C of the phage P2 (Webb *et al.*, 2004). Thus, we proposed to name the putative repressor protein in the prophage Pf4 of PAO1 as Pf4r (Pf4 repressor C) and in the prophage Pf5 in PA14 as Pf5r (Pf5 repressor C PA14_49020). Pf4r and Pf5r share 56% sequence identity. Between *pf4r* and *PA0717*, we found a putative ORF of 216 bp which encodes a protein of 71 aa. At a similar genomic location in the prophage Pf5, we found an ORF sharing 43.5% sequence identity with the one in Pf4. Thus, we named these ORFs as *xisF4* in Pf4 (Pf4 excisionase) and *xisF5* in Pf5 (Pf5 excisionase PA14_49010). XisF4 has a HTH DNA binding domain predicted by a BlastP search in the NCBI and IMG database, and shares 29% identity with the Xis of mycobacteriophage Pukovnik by structural similarity search using Phyre2 (Kelley *et al.*, 2015) (Fig. S1). Xis is required for Pukovnik prophage excision (Singh *et al.*, 2014), thus we hypothesized that XisF4 may function as an excisionase of Pf4.

### XisF4 and XisF5 promote prophage excision

**Figure 2:**
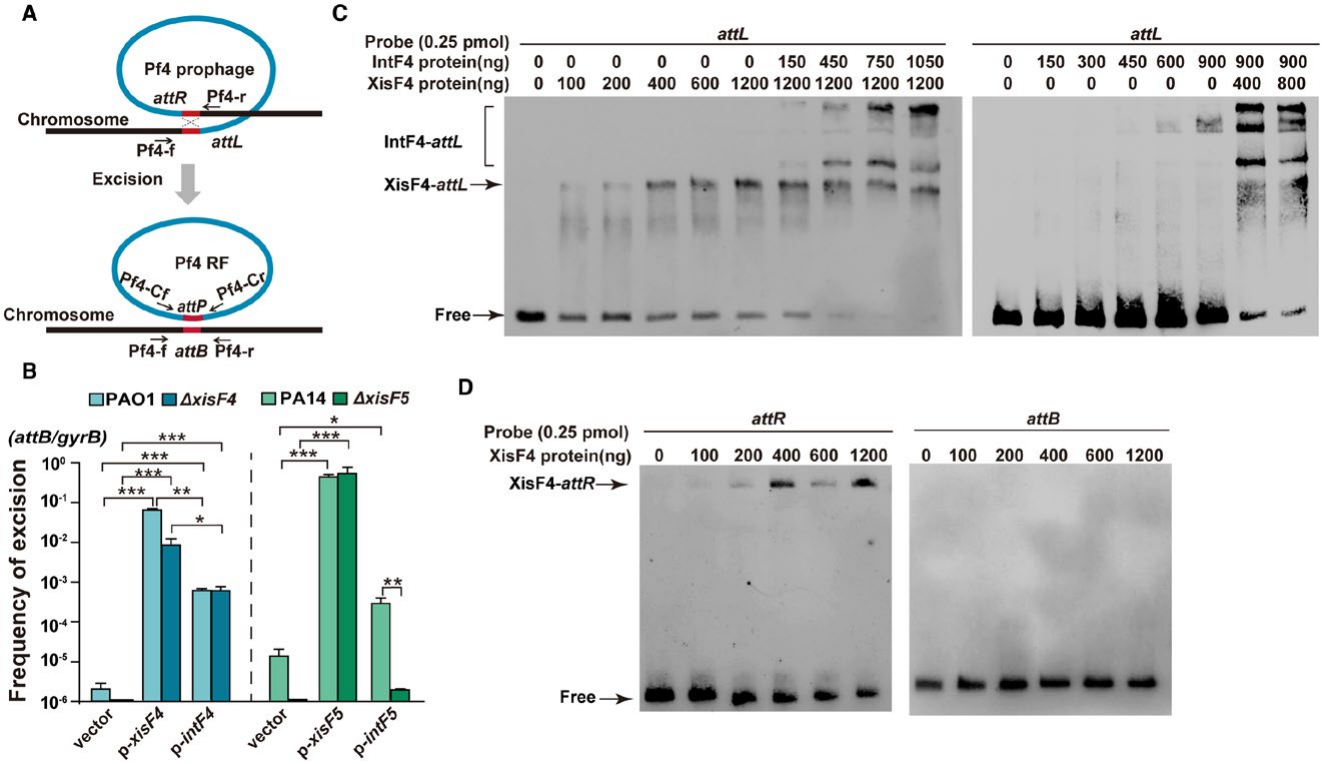
Integrase and excisionase in Pf4 and Pf5 promote prophage excision. A. A schematic diagram illustrates the excision of the prophage Pf4 from the PAO1 host chromosome and the formation of Pf4 replicative form (RF) molecules in the cytoplasm of PAO1. B. The frequency of Pf4 excision was quantified in PAO1 and Δ*xisF4 *overexpressing *intF4 *or *xisF4 *via pHERD20T‐based plasmids, and the frequency of Pf5 excision was quantified in PA14 and Δ*xisF5* overexpressing *intF5 *or *xisF5 *via pHERD20T‐based plasmids. Three independent cultures of each strain were used, and error bars indicate standard deviation. Unpaired two‐tailed Student’s *t* tests were used for statistical analysis; *p < *0.05 is marked *, *p* < 0.01 is marked **, and *p* < 0.001 is marked *** throughout the study. C. EMSA showed that XisF4 and IntF4 bound to *attL* in a concentration‐dependent manner. D. EMSA showed that XisF4 bound to *attR* in a concentration‐dependent manner but not to *attB*.

As far as we know, no prophage‐encoded excisionase have been characterized in filamentous prophages; thus, we first checked whether these newly identified genes encode functional excisionases. We cloned the predicted coding region of *xisF4* into pHERD20T to overexpress *xisF4 *using a P*_BAD_*inducible promoter in the PAO1 wild‐type and the Δ*xisF4* strains. qPCR using a forward primer (Pf4‐f) flanking the left attachment site and a reverse primer (Pf4‐r) flanking the right attachment site of the prophage Pf4 were employed to quantify the proportion of cells with Pf4 excised from the host chromosome (Fig. 2A). The frequency of Pf4 excision was very low in the PAO1 wild‐type during planktonic growth (approximately one out of 10^6^ cells), suggesting that the prophage Pf4 resides stably in the host genome (Fig. 2B). The frequency of Pf4 excision in PAO1 increased approximately 10^5^‐fold when *xisF4* was overexpressed via pHERD20T‐*xisF4 *in the PAO1 wild‐type strain, reaching up to ~1%. As expected, excision of Pf4 was undetectable (less than one out of 10^6^ cells) in the Δ*xisF4* strain, and it was restored by overexpressing *xisF4* (Fig. 2B). These results show that XisF4 can promote prophage excision. To confirm whether XisF4 is required for Pf4 excision, the frequency of Pf4 excision was also detected in the Δ*xisF4 *strain when the integrase IntF4 was overproduced. Although the ability of IntF4 to induce Pf4 excision in the Δ*xisF4* is lower than that of XisF4 under similar conditions, the frequency of Pf4 excision was similar in the PAO1 wild‐type strain and in the Δ*xisF4* strain when IntF4 was overproduced, suggesting that XisF4 is not required for Pf4 excision. Furthermore, similar results were obtained using PCR assays to detect Pf4 excision using a different pair of primers (Fig. S2). To further check the excisionase activity of XisF4, we performed electrophoretic mobility shift (EMSA) assays. As shown in Fig. 2C, XisF4 binds and shifts the attachment site of Pf4 prophage, and the presence of XisF4 also enhanced the binding of IntF4 to the attachment site. As a negative control, XisF4 could not bind or shift the bacterial attachment site (*attB*) once Pf4 is excised (Fig. 2D). These results show that XisF4 functions as an excisionase and can promote Pf4 excision. However, XisF4 is non‐essential for Pf4 excision as the integrase can still induce Pf4 excision in the absence of XisF4.

Next, we also investigated the function of the putative excisionase gene *xisF5 *and the putative integrase gene *intF5* (*PA14_48880*) in Pf5 prophage in the PA14 wild‐type strain. As expected, the frequency of Pf5 excision greatly increased (approximately 10^5^‐fold) when *xisF5 *was overexpressed via pHERD20T‐*xisF5* in the PA14 wild‐type strain (Fig. 2B). In addition, excision of Pf5 was undetectable in the Δ*xisF5* strain, and Pf5 excision can be restored by overexpressing *xisF5 *in the Δ*xisF5 *strain (Fig. 2B). To check whether XisF5 is required for Pf5 excision, the frequency of Pf5 excision was also detected in the Δ*xisF5* strain when the integrase was overproduced. Different from Pf4, no Pf5 excision was detected when the Pf5 integrase gene *intF5 *was overexpressed in the Δ*xisF5* strain. Additionally, Pf5 excision was also undetectable in the Δ*intF5 *strain even when *xisF5* was overexpressed (Fig. S3A). Furthermore, as expected, XisF5 and IntF5 can bind and shift *attL* of Pf5 as shown by EMSA (Fig. S3B). Taken together, we demonstrate that both excisionase and integrase in Pf prophages can promote prophage excision, and XisF5 is essential for Pf5 excision while XisF4 is non‐essential for Pf4 excision.

### XisF4 and XisF5 activate phage replication

**Figure 3:**
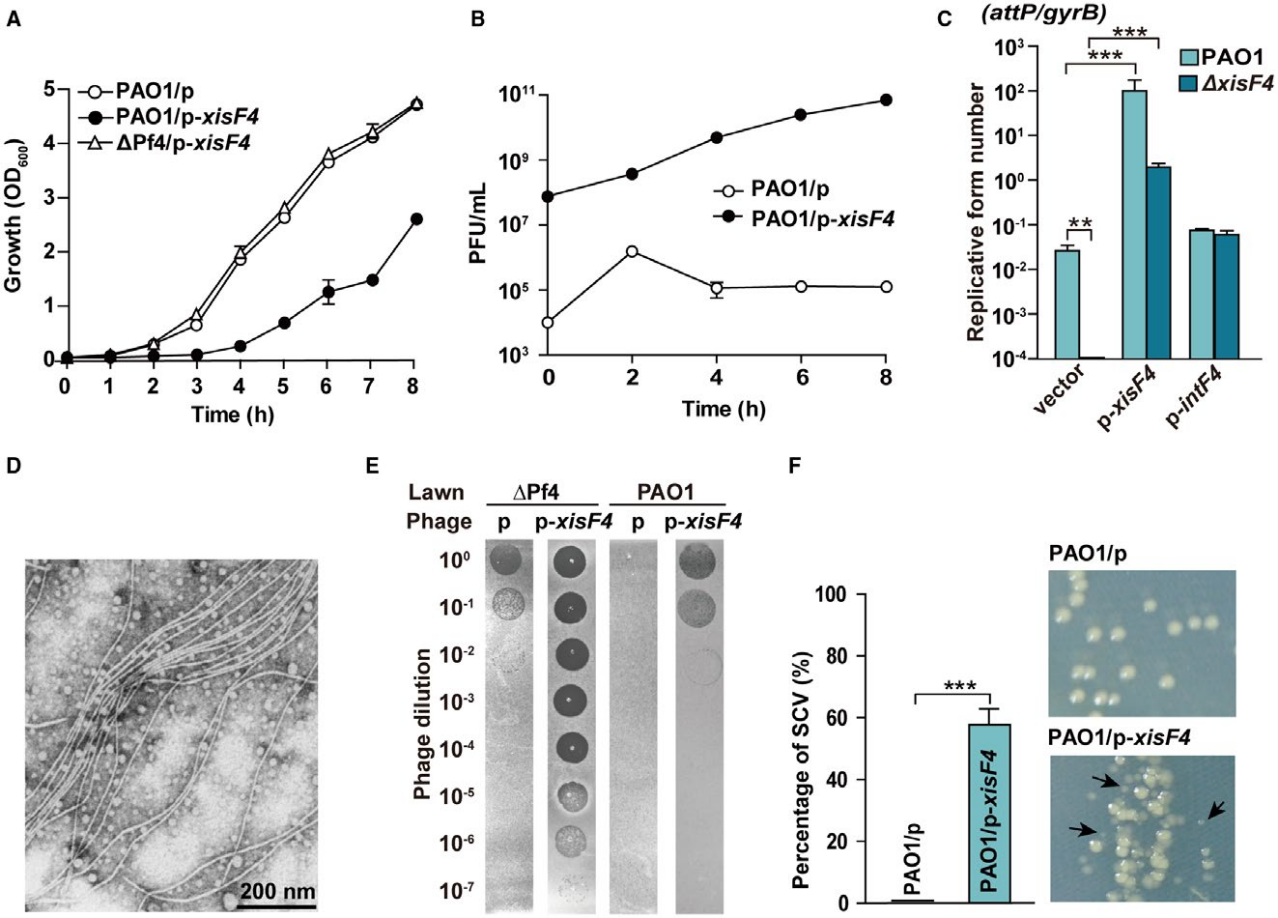
XisF4 activates Pf4 production. A. Growth (OD_600_) was tested in planktonically growing PAO1 carrying pHERD20T (p), PAO1 carrying pHERD20T‐*xisF4* (p‐*xisF4*) and ΔPf4 carrying pHERD20T‐*xisF4*; 10 mM arabinose was added at the beginning. B. Pf4 phage titers (PFU ml^–1^) were quantified on ΔPf4 lawns using supernatant from PAO1 carrying pHERD20T or pHERD20T‐*xisF4* under the same condition as shown in A. C. The number of Pf4 RF molecules were quantified in PAO1 and Δ*xisF4 *overexpressing *intF4 *or *xisF4*. D. Transmission electron microscopy (TEM) of Pf4 phage particles collected from the supernatant of PAO1 carrying pHERD20T‐*xisF4* at 4 h as shown in B. E. Plaque formation by the phage lysates at 4 h as shown in B. Phage lysates were serially diluted and 10 μl samples were dropped on PAO1 and ΔPf4 lawns, respectively. F. The percentage of SCVs (relatively small colonies formed) was calculated from PAO1 carrying pHERD20T or pHERD20T‐*xisF4* at 4 h as shown in B. Three independent cultures of each strain were used, and error bars indicate standard deviation.

We observed that overexpressing *xisF4* in the PAO1 wild‐type strain resulted in a severe growth inhibition. To test whether the growth inhibition is dependent on the presence of Pf4 prophage, we constructed a Pf4 deletion mutant strain (ΔPf4) in which the whole prophage was removed from PAO1 (Table 1, Fig. S5). As expected, growth inhibition was no longer detected when *xisF4* was overexpressed in the ΔPf4 strain (Fig. 3A). It has been shown that some excisionases such as Cox protein of P2 phage can function as a transcriptional regulator in addition to as an excisionase or RDF (Saha *et al.*, 1989; Yu and Haggard‐Ljungquist, 1993). Thus, we tested whether XisF4 can regulate the production of Pf4 phage particles. Active Pf4 phage particles produced by PAO1/pHERD20T‐*xisF4* were quantified by applying the supernatant collected from this strain onto the bacterial lawn formed by the ΔPf4 strain. Notably, Pf4 phage particles increased approximately 10^6^‐fold when *xisF4 *was overexpressed, reaching up to 10^11^ PFU ml^–1^ (Fig. 3B), indicating that XisF4 increases phage production. Replication of filamentous phages involves the generation of double‐stranded DNA known as replicative form (RF) molecules (Marvin, 1998). Thus, we further explored whether XisF4 can promote the formation of Pf4 RF using qPCR. An inner forward primer near the left attachment site (Pf4‐Cr) and an inner reverse primer near the right attachment site (Pf4‐Cf) of the prophage Pf4 were then employed to quantify the number of Pf4 RF molecules in the mixed population using qPCR (Fig. 3C). Overexpressing *xisF4* via pHERD20T‐*xisF4 *in the PAO1 wild‐type strain greatly increased the number of Pf4 RF molecules by approximately 10^4^‐fold, while Pf4 RF was undetectable in the Δ*xisF4* strain (Fig. 3C). In contrast, although IntF4 can also increase prophage excision, overproducing IntF4 via pHERD20T‐*intF4 *did not affect the number of Pf4 RF (Fig. 3C). Similar results were obtained for *xisF5 *in Pf5 prophage in PA14 strain. Overexpressing *xisF5* via pHERD20T‐*xisF5 *in the PA14 wild‐type strain greatly increased the number of Pf5 RF molecules by approximately 10^4^‐fold, while overexpressing *intF5* did not affect the number of Pf5 RF (Fig. S4).

**Table 1:** Bacterial strains and plasmids used in this study.

10	Description	Source
**Strains**		
***Escherichia coli***		
DH5α	F^–^ *φ80lacZ∆M15 ∆(lacZYA‐argF)U169 recA1 endA1 hsdR17(rk^–^*, *mk^+^)phoA supE44 thi‐1 gyrA96 relA1 tonA*	Novagen
K‐12 BW25113	*lacI* ^q^ *rrnB*_T14 _Δ*lacZ*_WJ16 _ *hsdR*514 Δ*araBAD*_AH33 _Δ*rhaBAD*_LD78_	Baba *et al*. (2006)
BL21(DE3)	F*^– ^ompT hsdS_B_(r_B_^–^m_B_^–^) gal dcm λ*(DE3) Ω P_tacUV5_::T7 polymerase	Novagen
***P. aeruginosa***		
PAO1	Wild type	Stover *et al*. (2000)
Δ*pf4r*	*pf4r* deletion mutant derived from PAO1	This study
Δ*xisF4*	*xisF4* deletion mutant derived from PAO1	This study
ΔPf4	whole Pf4 prophage removed from PAO1 host chromosome	This study
PA14	Wild type	Liberati *et al*. (2006)
Δxis*F5*	*xisF5* deletion mutant derived from PA14	This study
Δ*intF5*	*intF5* deletion mutant derived from PA14	This study
Δ*mvaT*	*mvaT *deletion mutant derived from PAO1	This study
Δ*mvaU*	*mvaU* deletion mutant derived from PAO1	This study
Δ*mvaU*Δ*mvaT*	*mvaT* and *mvaU *double deletion mutant derived from PAO1	This study
PAO1::P*_pf4r_‐lacZ*	LacZ reporter strain	This study
ΔPf4::P*_pf4r_‐lacZ*	LacZ reporter strain	This study
PAO1:: P*_xisF4_‐lacZ*	LacZ reporter strain	This study
ΔPf4:: P*_xisF4_‐lacZ*	LacZ reporter strain	This study
PAO1::P*_PA0720_‐lacZ*	LacZ reporter strain	This study
PAO1::P*_PA0724_‐lacZ*	LacZ reporter strain	This study
PAO1::P*_PA0727_‐lacZ*	LacZ reporter strain	This study
**Plasmids**		
pHERD20T	Ap^R^, expression vector with araC‐P_BAD _promoter	Qiu *et al*., (2008)
pHERD20T‐*xisF4*	Ap^R^, *xisF4* in pHERD20T EcoRI/HindIII	This study
pHERD20T‐*pf4r*	Ap^R^, *pf4r* in pHERD20T EcoRI/HindIII	This study
pHERD20T‐*intF4*	Ap^R^, *intF4* in pHERD20T EcoRI/XbaI	This study
pHERD20T‐*xisF5*	Ap^R^, *xisF5* in pHERD20T EcoRI/HindIII	This study
pHERD20T‐*pf5r*	Ap^R^, *pf5r* in pHERD20T EcoRI/HindIII	This study
pHERD20T‐*intF5*	Ap^R^,* intF5* in pHERD20T EcoRI/XbaI	This study
pET28b	Km^R^, expression vector	Novagen
pET28b‐*intF4*	Km^R^, *intF4 *in pET28b NcoI/HindIII	This study
pET28b‐*xisF5*	Km^R^, *xisF5* in pET28b NcoI/HindIII	This study
pET28b‐*intF5*	Km^R^, *intF5* in pET28b NcoI/HindIII	This study
pEX18AP	Ap^R^, *oriT* ^+^, *sacB* ^+^, gene replacement vector	Hoang *et al*. (1998)
pFLP2	Ap^R^, Flp recombinase‐expressing plasmid	Hoang *et al*. (1998)
pPS856	Ap^R^, Gm^R^; for amplifying gentamycin resistance cassette	Hoang *et al*. (1998)
pEX18AP‐*pf4r*‐up‐GM‐down	Gm^R^, Car^R^, for deleting *pf4r*	This study
pEX18AP‐*xisF4*‐up‐GM‐down	Gm^R^, Car^R^, for deleting *xisF4*	This study
pEX18AP‐Pf4‐up‐GM‐down	Gm^R^, Car^R^, for deleting Pf4	This study
pEX18Ap‐*mvaT‐*up‐GM‐down	Gm^R^, Car^R^, for deleting *mvaT*	This study
pEX18Ap‐*mvaU*‐up‐GM‐down	Gm^R^, Car^R^, for deleting *mvaU*	This study
mini‐CTX‐*LacZ*	Tet^R^, integration vector for single‐copy, chromosomal *lacZ* fusions; Ω‐FRT‐*attP*‐MCS, *ori*, *int*, and *oriT*	Becher and Schweizer (2000)
pCTX‐P*_pf4r_*‐*lacZ*	Tet^R^, −313 bp relative to translational start site of *pf4r *cloned into mini‐CTX‐*lacZ*	This study
pCTX‐ P*_xisF4_‐lacZ*	Tet^R^, −300 bp relative to translational start site of *xisF4 *cloned into mini‐CTX‐*lacZ*	This study
pCTX‐P*_PA0720_*‐*lacZ*	Tet^R^, −345 bp relative to translational start site of *PA0720 *cloned into mini‐CTX‐*lacZ*	This study
pCTX‐P*_PA0724_*‐*lacZ*	Tet^R^, −334 bp relative to translational start site of *PA0724 *cloned into mini‐CTX‐*lacZ*	This study
pCTX‐P*_PA0727_*‐*lacZ*	Tet^R^, −360 bp relative to translational start site of *PA0727 *cloned into mini‐CTX‐*lacZ*	This study

A large number of filamentous phage particles were also observed using electron microscopy in the supernatant of PAO1 when *xisF4 *was overexpressed (Fig. 3D). In addition, we found that high titers of Pf4 (> 10^8^ PFU ml^–1^) can not only infect the ΔPf4 strain but can also re‐infect the PAO1 wild‐type strain that contains an integrated copy of the same phage (Fig. 3E). Previous work showed, by using the flow‐cell biofilm assay, that the formation of small colony variants (SCVs) was induced at the late stage of PAO1 biofilm development (Rice *et al.*, 2009). Here, the occurrence of SCVs increased up to 55% when *xisF4 *was overexpressed via PAO1/pHERD20T‐*xisF4*, while SCVs were rarely detected in PAO1/pHERD20T in planktonic growth conditions (Fig. 3F). These results collectively demonstrate that XisF4 and XisF5 can activate phage replication.

### XisF4 promotes Pf4 replication by activating PA0727

**Figure 4:**
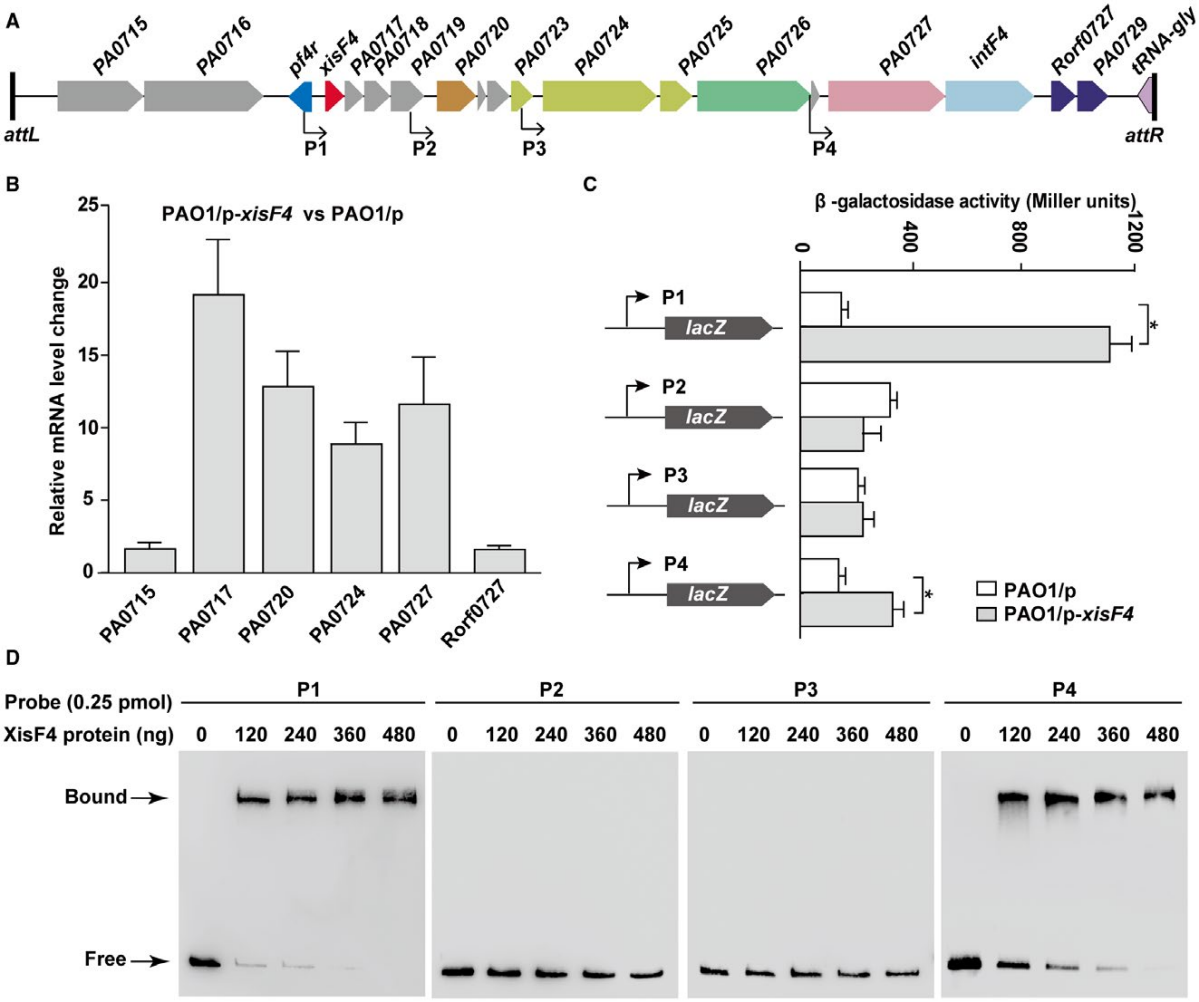
XisF4 induces the expression of *PA0727*. A. Start sites of four putative promoter regions of the related operons in Pf4 are indicated with arrows, with P1 for *xisF4*‐*PA0719*, P2 for *PA0720*‐*0723*, P3 for *PA0724‐0726 *and P4 for *PA0727‐0728*. B. Relative mRNA levels of the first gene (except *PA0717*) of the putative operons shown in A in PAO1/pHERD20T‐*xisF4* *versus *PAO1/pHERD20T. C. The *lacZ* reporter activities were determined in strain PAO1 carrying pHERD20T and pHERD20T‐*xisF4*. When cells were grown to OD_600 _~ 0.1, 10 mM arabinose was added for 3 h of induction. Three independent cultures were used, and error bars indicate standard deviation in B and C. D. EMSA showed that XisF4 bound to the promoter regions of P1 and P4 in a concentration‐dependent manner, but XisF4 did not bind to the promoter regions of P2 or P3.

To explore how XisF4 regulates phage production, we first performed qRT‐PCR to check the expression of Pf4 genes by overexpressing *xisF4* via pHERD20T‐*xisF4* with 10 mM arabinose for 30 min. The structural genes conserved in Pf4 and Pf5 (from *PA0717 *to *PA0726*) were all upregulated by 10~20‐fold when *xisF4* was overexpressed (Fig. 4AB). In addition, *PA0727*, which encodes the replication initiator protein, was also highly induced. In contrast, the genes upstream and downstream of *PA0717‐intF4* were only slightly induced (< 2‐fold), likely due to the presence of more copies of Pf4 RF (Fig. 4B). These results suggest that genes encoding phage structural gene and phage replication initiator were specifically activated when *xisF4* was overexpressed. To gain further insights into the regulation of XisF4 on Pf4 genes, four different chromosomal *lacZ* transcriptional fusions (P*_xisF4_‐lacZ*, P*_PA0720_*‐*lacZ*, P*_PA0724_*‐*lacZ* and P*_PA0727_*‐*lacZ*) (Table 1) were constructed to test the promoter activity of the four operons *in vivo* by measuring β‐galactosidase activity. When *xisF4* was overexpressed in PAO1 with the addition of 10 mM arabinose (at OD_600_ ~ 0.1) for 3 h, the promoter activity of *xisF4 *(P*_xisF4_‐lacZ*) increased 11 ± 1‐fold, and the promoter activity of *PA0727* (P*_PA0727‐_lacZ*) increased 2.2 ± 0.3‐fold (Fig. 4C). However, overexpressing *xisF4* did not affect the promoter activities of *PA0720* and *PA0724 *(Fig. 4C). Furthermore, electrophoretic mobility shift (EMSA) assays showed that XisF4 bound and shifted its own promoter and the promoter region of *PA0727* but not the promoter regions of *PA0720 *or *PA0724* (Fig. 4D). Taken together, these results demonstrate that XisF4 positively regulates *PA0727* and that overproduction of XisF4 greatly increases Pf4 phage production.

### Pf4r confers immunity to Pf4

**Figure 5:**
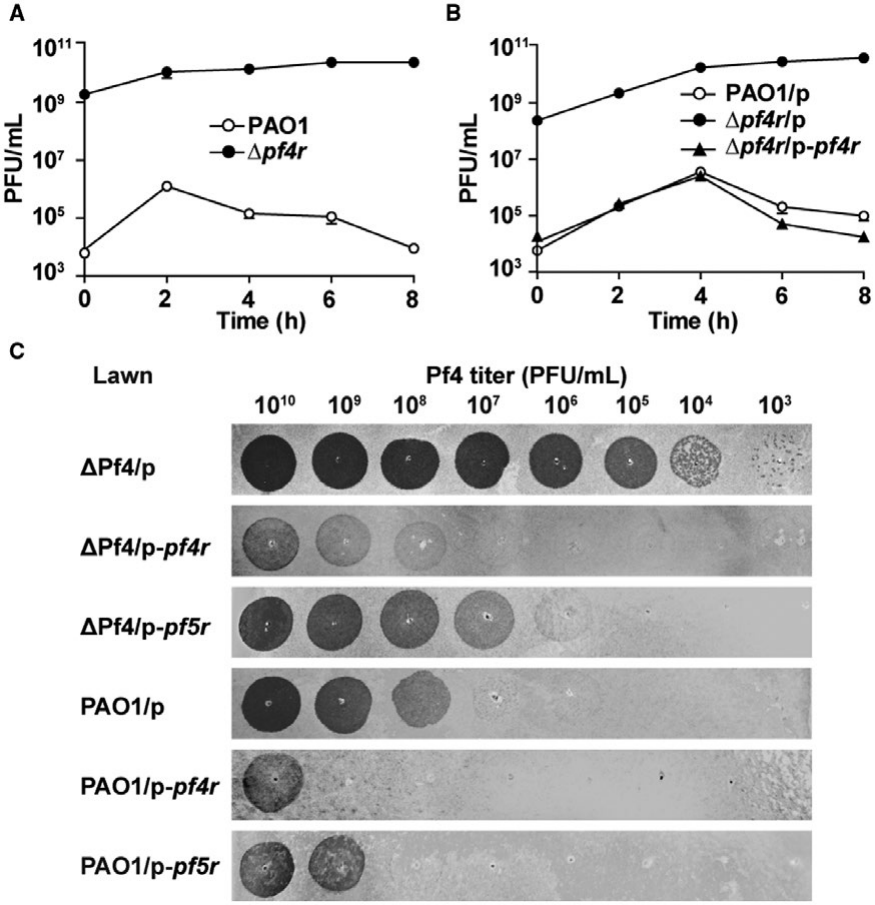
Pf4r confers immunity to Pf4. A. Pf4 phage titers (PFU ml^–1^) were quantified on ΔPf4 lawn using culture supernatant from planktonically growing PAO1 and Δ*pf4r* strains at different times. B. Pf4 phage titers (PFU ml^–1^) were quantified on ΔPf4 lawns using culture supernatant from planktonically growing PAO1 carrying pHERD20T, Δ*pf4r* carrying pHERD20T and Δ*pf4r* carrying pHERD20T‐*xisF4*. At the beginning of the culture, 10 mM arabinose was added to induce the expression of *xisF4*. C. Pf4 phages were collected from planktonic culture supernatant of PAO1 with overexpression of XisF4. Serially diluted phages were then applied to lawns of ΔPf4 carrying pHERD20T, pHERD20T‐*pf4r *and pHERD20T‐*pf5r*, and lawns of PAO1 carrying pHERD20T, pHERD20T‐*pf4r *and pHERD20T‐*pf5r*. Three independent cultures of each strain were used, and error bars indicate standard deviation in A and B.

Pf4r in Pf4 shares 42% homology with the repressor C of the phage P2 (Webb *et al.*, 2004). Repressor C in P2 controls the lytic conversion of P2 prophage (Saha *et al.*, 1987b); thus, we first tested whether Pf4r can control the lysogenic conversion of Pf4. We constructed a *pf4r* deletion‐mutant strain (Δ*pf4r*) in PAO1 (Table 1). Pf4 phage production was quantified by applying the supernatant collected from the planktonic culture of the Δ*pf4r* and the PAO1 wild‐type strains to the bacterial lawn formed by the ΔPf4 strain. As expected, deletion of *pf4r* increased Pf4 production 10^5^‐fold compared to the PAO1 wild‐type strain (Fig. 5A). Furthermore, complementation of *pf4r* via pHERD20T‐*pf4r *in the Δ*pf4r* strain greatly reduced Pf4 production (Fig. 5B), suggesting that Pf4r functions as a repressor for Pf4 phage production. In lambdoid, CTXφ and P2 phages, phage repressors are known to mediate phage immunity (Wilgus *et al.*, 1973; Kimsey and Waldor, 1998). To explore the role of Pf4r in Pf4 immunity, we used Pf4 phages to infect the ΔPf4 strain in the absence of *pf4r* (carrying pHERD20T) and in the presence of *pf4r* (carrying pHERD20T‐*pf4r*). As expected, the ability of Pf4 to infect the ΔPf4 strain decreased 10^6^‐fold when Pf4r was overproduced via pHERD20T‐*pf4r *(Fig. 5C). Additionally, overproduction of Pf4r via pHERD20T‐*pf4r* in the PAO1 wild‐type strain reduced the ability of Pf4 to re‐infect PAO1 approximately 100‐fold, and overproduction of Pf5r via pHERD20T‐*pf5r* in the PAO1 wild‐type strain also reduced the ability of Pf4 phages to re‐infect PAO1 approximately 10‐fold (Fig. 5C). These results collectively demonstrate that Pf4r and Pf5r both confer immunity to Pf4 infection.

### Pf4r auto‐activates itself and represses *xisF4*

**Figure 6:**
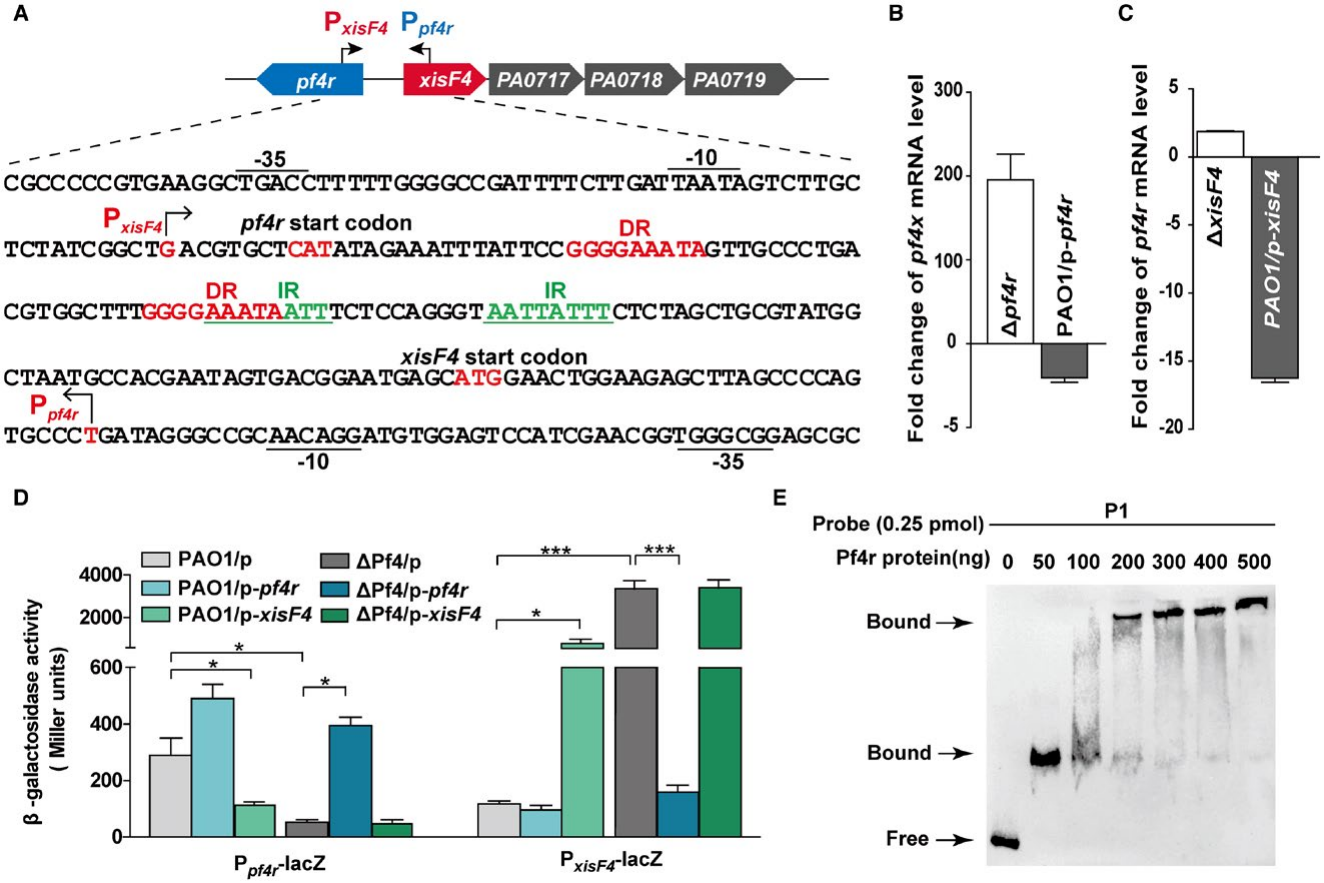
Pf4r represses *xisF4* while activating itself. A. A schematic diagram indicates the intergenic region between *xisF4* and *xisF5*. The transcriptional start sites of *xisF4 *and *pf4r* were determined by 5′‐RACE, and arrows indicate the direction of transcription. DR and IR indicate directed and inverted repeats, respectively. B. Fold change of mRNA levels of *xisF4* in Δ*pf4r* *versus* PAO1, and in PAO1/pHERD20T‐*pf4r* *versus* PAO1/pHERD20T. C. Fold change of mRNA levels of *pf4r* in Δ*xisF4 versus* PAO1, and in PAO1/pHERD20T‐*xisF4 versus* PAO1/pHERD20T. D. The β‐galactosidase activity of P*_pf4r_*_–_ *_lacZ_* and P*_xisF4_*_–_ *_lacZ_* were determined in PAO1 and ΔPf4 carrying pHERD20T, pHERD20T‐*pf4r* and pHERD20T‐*xisF4*, respectively. 10 mM arabinose was added for induction for 3 h at OD_600 _~ 0.1. Three independent cultures of each strain were used, and error bars indicate standard deviation in B, C and D. E. EMSA showed that Pf4r bound to the promoter region of P1 (as shown in Fig. A) in a concentration‐dependent manner.

Previous analysis predicted that the upstream region of the structural gene *PA0717* is the regulatory region of Pf4 (McElroy *et al.*, 2014). Since the new ORF (XisF4) identified in this study is located between *PA0717* and *pf4r*, 5′‐RACE was employed to determine the transcriptional start sites of *pf4r* and *xisF4*, respectively. As shown in Fig. 6A, the transcriptional start site of *xisF4* is 129 bp upstream of the translational start site of *xisF4 *and 11 bp downstream of that of *pf4r*. On the other hand, the transcriptional start site of *pf4r* is 150 bp upstream of the translational start site of *pf4r *and 31 bp downstream of that of *xisF4.* The 5′‐untranslated regions of the *pf4r *and *xisF4 *transcripts overlap and contain a directed repeat (DR, 5′‐GGGGAAATA‐3′) and an inverted repeat (IR, 5′‐AATTATTT‐3′) (Fig. 6A).

To check the cross‐regulation between Pf4r and XisF4, expression of *xisF4* in the presence *versus *in the absence of *pf4r *was assessed by qRT‐PCR. The level of *xisF4* transcript was induced 196 ± 32‐fold in the Δ*pf4r* strain compared with the PAO1 wild‐type strain, and the level of *xisF4* transcript was repressed 2.9 ± 0.9‐fold when *pf4r *was overexpressed via pHERD20T‐*pf4r *compared with the empty plasmid in PAO1 (Fig. 6B). Similarly, expression of *pf4r* in the presence *versus *in the absence of *xisF4 *was also assessed. The level of *pf4r* transcript was repressed 16.2 ± 0.7‐fold when *xisF4 *was overexpressed via pHERD20T‐*xisF4 *compared with the empty plasmid in the PAO1 strain (Fig. 6C). However, the level of *pf4r* transcript was not changed in the Δ*xisF4* strain compared with the PAO1 wild‐type strain. These results showed that Pf4r represses the expression of *xisF4* and vice versa. Furthermore, these results also suggested that *xisF4* was repressed while *pf4r *was expressed in the PAO1 wild‐type strain to ensure lysogeny under normal conditions. To further determine how Pf4r regulates *xisF4 *and *pf4r*, two transcriptional *lacZ* fusions, one containing the *xisF4 *promoter (P*_xisF4_*‐*lacZ*) and the other containing the *pf4r* promoter (P*_pf4r_*‐*lacZ*), were constructed and integrated into the PAO1 wild‐type strain and the ΔPf4 strain, respectively. Noticeably, the promoter activity of *pf4r *was 5.2 ± 0.2‐fold higher in the PAO1 wild‐type strain (PAO1/pHERD20T) compared with the ΔPf4 strain (ΔPf4/pHERD20T). In contrast, the promoter activity of *xisF4 *was 28 ± 1‐fold higher in the ΔPf4 strain (ΔPf4/pHERD20T) compared with the PAO1 wild‐type strain (PAO1/pHERD20T). Corroborating the above qRT‐PCR results, the promoter activity of *pf4r* decreased 3.0 ± 0.5‐fold when *xisF4 *was overexpressed in the PAO1 wild‐type strain and increased 7.0 ± 0.3‐fold when *pf4r *was overexpressed in the ΔPf4 strain (Fig. 6D). These results demonstrate that XisF4 repressed the expression of *pf4r* and Pf4r activated itself in the absence of *xisF4*. Similarly, the promoter activity of *xisF4 *reduced 21 ± 1‐fold when *pf4r *was overexpressed in the ΔPf4 strain and increased 11 ± 1‐fold when *xisF4 *was overexpressed in the PAO1 wild‐type strain (Fig. 6D). These results demonstrated that Pf4r repressed the expression of *xisF4* and XisF4 can activate itself in the presence of *pf4r*. Furthermore*,* EMSA was performed to test the DNA binding activity of Pf4r to the overlapped promoter regions of *pf4r *and *xisF4*. As expected, Pf4r bound and shifted this region (Fig. 6E) and XisF4 also bound and shifted the same region as shown above (Fig. 4D). Taken together, these results collectively show that Pf4r represses the transcription of *xisF4* and XisF4 represses the expression of *pf4r*.

### MvaT/MvaU control *xisF4 *transcription

**Figure 7:**
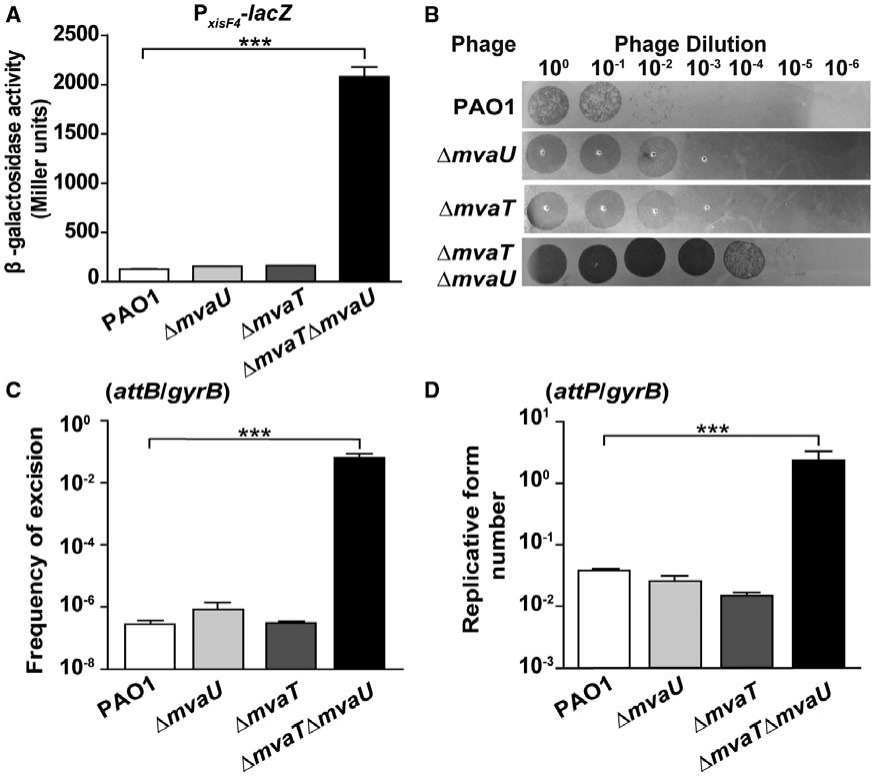
MvaT and MvaU coordinately repress *xisF4*. A. The β‐galactosidase activity of the P*_xisF4_*_–_ *_lacZ_* reporter was determined in PAO1, Δ*mvaT*, Δ*mvaU* and Δ*mvaT*Δ*mvaU*. B. Phages were collected from planktonic culture (OD_600_ ~ 1.0) supernatants of PAO1, Δ*mvaT*, Δ*mvaU* and Δ*mvaT*Δ*mvaU*. Serial dilutions were applied to lawns of ΔPf4. C. The frequency of Pf4 excision was quantified in PAO1, Δ*mvaT* and Δ*mvaU* and Δ*mvaT*Δ*mvaU*. D. The numbers of Pf4 RF molecules were quantified in PAO1, Δ*mvaT*, Δ*mvaU* and Δ*mvaT*Δ*mvaU*. Three independent cultures were used, and error bars indicate standard deviation.

Our earlier work revealed that H‐NS controls P4‐like excisionase gene *alpA* in *Shewanella oneidensis* and Hha controls P4‐like excisionase gene *alpA* in *E. coli* K‐12 (Wang *et al.*, 2009; Zeng *et al.*, 2016). In PAO1, two H‐NS family proteins, MvaT and MvaU, function coordinately as xenogeneic silencers (Castang *et al.*, 2008). A previous ChIP‐chip assay also suggested that one of the binding regions of MvaT and MvaU is located between *PA0715* and *PA0717* (Castang and Dove, 2012). Here, to investigate whether the H‐NS family protein regulates *xisF4 *in PAO1, two single‐deletion strains, each lacking one of the H‐NS family genes, and a double‐deletion strain lacking two H‐NS family genes were constructed (Table 1). Next, a chromosomal *lacZ* transcriptional fusion of the *xisF4* promoter (P*_xisF4_‐lacZ*) was integrated into these deletion mutants to test the promoter activity of *xisF4* *in vivo* by measuring β‐galactosidase activity. The promoter activity of *xisF4 *increased 16 ± 1‐fold in the Δ*mvaT*Δ*mvaU* double‐mutant strain compared with the wild‐type PAO1 strain (Fig. 7A). However, the promoter activity of *xisF4* in the Δ*mvaT* strain or in the Δ*mvaU* strain was similar to that in the wild‐type PAO1 strain (Fig. 7A). Furthermore, deleting both H‐NS family proteins also greatly induced Pf4 production (10^3^‐fold), Pf4 excision (10^5^‐fold) and the number of Pf4 RF molecules (10^2^‐fold) (Fig. 7B–D). Taken together, these results suggest that the host proteins MvaT and MvaU coordinately repress the production of Pf4 by directly repressing *xisF4 *transcription.

### XisF4 and XisF5 represent two major subfamilies of excisionases in Pf prophages

**Figure 8:**
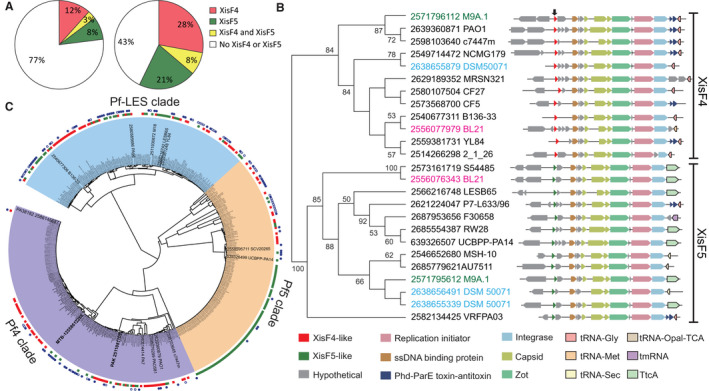
XisF4 and XisF5 are prevalent in Pf Phage. A. The left pie chart shows the proportions of the strains carrying XisF4 (red), XisF5 (green) or both (yellow) among 1999 *Pseudomonas* strains. The right pie chart shows the proportions of the strains carrying XisF4, XisF5 or both among 696 *Pseudomonas* *aeruginosa* strains. B. Neighbor‐joining tree of excisionases showing the phylogenetic relationships of the excisionase homologues in *P. aeruginosa *strains. Bootstrap values greater than 50% are indicated at the nodes. The name on each node indicates the Gene ID and the strain name in the IMG/M database, and the excisionase homologues from the same strain are marked by the same font color. The arrow on the top shows the position of the excisionase. The regions neighboring these excisionase genes were analyzed and are shown for each node. C. Phylogenetic tree of PA0724 (minor coat protein) homologues indicates the relationship of the Pf prophages. Three clades are indicated by color coding: purple‐Pf4, orange‐Pf5 and blue‐Pf‐LES. The first outer ring represents the presence of XisF4 (red), XisF5 (green) or no XisF4 or XisF5 (blank) in the specific Pf phage carrying the corresponding PA0724 homologue at each node. The second outer ring indicates that the strain at each node has multiple copies of PA0724 homologues located in a complete Pf phage; a filled blue circle indicates two copies, and a blue circle indicates more than two copies.

To investigate the prevalence of XisF4 and XisF5 among *Pseudomonas* species, we first set a gene profile within the genus *Pseudomonas* in IMG/M database with the minimal identity of 60% and *e*‐value of 0.01 (Mukherjee *et al.*, 2017). Among 1999 sequenced *Pseudomonas *strains, 233 of them carry a XisF4 homologue, 165 of them carry a XisF5 homologue and 62 of them carry both XisF4 and XisF5 homologues (Fig. 8A). Among 696 sequenced *P. aeruginosa* strains deposited in the IMG/M database*,* 194 carry a XisF4 homologue, 146 carry a XisF5 homologue and 58 carry homologues of both XisF4 and XisF5 (Fig. 8A). We next investigated the phylogenetic relationships among the XisF4 or XisF5 homologues. Notably, we found that the excisionase genes were clustered into two distinct groups, the XisF4 group and the XisF5 group (Fig. 8B). Importantly, overexpressing *xisF5* in PAO1 was unable to induce Pf4 excision, and vice versa (Fig. S6), suggesting these two groups might be functionally divergent. To further determine the prevalence of excisionases among Pf prophages, we first analyzed the abundance and diversity of Pf prophages in *P. aeruginosa*. Considering that the phylogenetic relationship of phages can be represented by their conserved coat proteins (Kauffman *et al.*, 2018), a tree based on the minor coat protein (PA0724) of Pf prophages (carrying the conserved region PA0720‐PA0727) was constructed. According to the phylogenetic tree, Pf prophages were clustered into three clades, Pf4, Pf5 and Pf‐LES (Fig. 8C), which is similar to the previous phylogenetic analysis of Pf prophages based on whole‐genome analysis (four clades: Pf4, Pf5 Pf7, and Pf‐LES) (Knezevic *et al.*, 2015). The Pf4 group obtained based on minor coat proteins contains the Pf7 clade, including the prophages of strains PA7, PA38182 and MTB‐1t. Among the 433 Pf phages, 247 (57%) contain a XisF4 or XisF5 excisionase (Fig. 8C, Table S2), suggesting that more than half of the Pf phages are integrated into the host chromosome instead of replicating episomally. Our analysis also revealed that many *P. aeruginosa* strains carry multiple copies of Pf phages belonging to the same or different clades (Fig. 8C). These multiple copies of Pf prophages are integrated into different tRNA genes, which are matched to the different subfamilies of integrase based on phylogenetic analysis (Figs 8C and S7). Taken together, these analyses indicate that XisF4 and XisF5 are widespread among Pf phages in *P. aeruginosa*.

## Discussion

In this study, we report for the first time that filamentous phages encode their own excisionases. More importantly, we demonstrate that the two excisionases (XisF4 in Pf4 and XisF5 in Pf5) not only promote Pf prophage excision but also activate prophage replication. To our knowledge, due to their relatively small genome sizes (< 12 kb), filamentous phages usually do not encode their own excisionases. The identification of excisionases in Pf4 and Pf5 prophages fundamentally extends the current understanding of genome excision and host regulation of filamentous phages. Specifically, we show that high expression of *pf4r* is needed to ensure the lysogeny of PAO1 under normal conditions while high expression of *xisF4* is needed to induce prophage excision and replication under specific conditions. XisF4 and XisF5 share medium sequence identity, and our results demonstrate that XisF5 is essential for Pf5 excision while XisF4 is non‐essential for Pf4 excision. In addition, phylogenetic analysis demonstrated that XisF4 and XisF5 represent the two subfamilies of excisionase of Pf phages in *P. aeruginosa* strains, and they are highly prevalent in Pf prophages. Thus, prophage excision regulated by prophage‐encoded excisionase should be common for Pf prophages in *P. aeruginosa* strains. Also, our results suggest that using genetically modified excisionase to induce prophage excision without regulating phage replication would be a feasible way to remove integrated filamentous prophage from the *Pseudomonas* host genome.

The genome sizes of Pf1, Pf4 and Pf5 are 7.3 kb, 12.4 kb and 10.6 kb, respectively (Hill *et al.*, 1991). Comparative analysis revealed that both Pf4 and Pf5 exhibit mosaic phage genome structures. Most of the structural genes of Pf1, Pf4 and Pf5 are highly conserved, except for the minor coat proteins g3p (PA0724 in Pf4, PA14_48930 in Pf5 and ORF437 in Pf1) which share a lower sequence similarity. The minor coat protein g3p is involved in the initial step of phage infection by interacting with host pilus (Holland *et al.*, 2006), and the low sequence identity in minor coat proteins suggest that these Pf prophages have been divergent and that there has been a long co‐evolution of the bacteria‐host interaction (Marvin *et al.*, 2014). Apart from the backbone shared by these three Pf prophages, Pf4 and Pf5 also carry two regulatory genes, repressor *C* (*pf4r* and *pf5r*) and excisionase (*xisF4* and *xisF5*). In this study, we demonstrate that XisF4 acts in three ways: acting as a transcriptional repressor of *pf4r*, acting as a transcriptional activator of PA0727 (which is the replication initiator of Pf4), and promoting the excision of Pf4. The Cox protein of P2 also has similar three functions; it represses the promoter of repressor gene, activates the gene which controls phage replication, and promotes the excision of P2 (Saha *et al.*, 1987a; 1989; Eriksson and Haggard‐Ljungquist, 2000). P2 is a major family of temperate dsDNA phages and is different from filamentous phages in gene arrangement and morphology (Bertani and Bertani, 1971; Crowther, 1980). Although they are functionally equivalent, there is no sequence similarity between Cox and XisF4 or XisF5. Instead, XisF4 shares 30% similarity with a putative AlpA excisionase of P4‐like prophage of *Pseudomonas mandelii *(Fig. S1). P4 is a satellite phage that relies on P2 as a helper to supply the gene products necessary for phage particle assembly and cell lysis (Barrett *et al.*, 1976). Interestingly, repressor C in Pf4 shows 42% sequence identity with the repressor C of phage P2 (Webb *et al.*, 2004). Moreover, apart from the conserved regulatory genes in both Pf4 and Pf5, there are several unique genes in Pf4 and Pf5. For example, the Phd‐ParE toxin‐antitoxin system, a putative ATPase and a reverse transcriptase are found in Pf4 but not in Pf5 or Pf1 (Webb *et al.*, 2004). Pf5 has a unique gene encoding a protein with a predicated ParA nucleotide‐binding domain (Mooij *et al.*, 2007). Further studies are under way to explore the functions of these unique phage genes.

It has been previously reported that Pf4 phages released from PAO1 biofilms can re‐infect the PAO1 wild‐type that contains an integrated copy of the same phage (Rice *et al.*, 2009). Further sequencing results revealed that mutations within or upstream of repressor *c* gene of Pf4 were accumulated in the ‘superinfective’ Pf4 phages released from PAO1 flow‐cell biofilms (McElroy *et al.*, 2014). Our analysis showed that these mutations are located at the upstream region of the *xisF4* gene. Furthermore, we demonstrate that the repressor C in Pf4 and Pf5 confer immunity to Pf4 infection and Pf4 phages released from PAO1 overexpressing *xisF4* were also capable of re‐infecting PAO1 wild‐type strain. However, these phages can only re‐infect PAO1 at high titers (> 10^8^ PFU ml^–1^) and this ability of re‐infecting PAO1 is further reduced by overexpressing *pf4r* or *pf5r* in PAO1. These results suggest that a high amount of phage particles might circumvent the phage immunity conferred by the phage repressor protein. Nevertheless, it remains to be determined whether and how the excisionase and/or the repressor C directly contribute to the emergence of superinfective Pf4 phages in PAO1 flow‐cell biofilms.

For lambda phage, it is clear that two host‐encoded proteins, RecA and LexA, play an important role in the control of the lysis‐lysogeny switch. However, very few studies directly focused on the lysis‐lysogeny switch of Pf prophages. Nevertheless, activation of genes in Pf prophages in *P. aeruginosa* have been reported in several stressed conditions. Pf prophage‐encoded genes were found to be strongly upregulated in *P. aeruginosa* biofilm cells using DNA microarrays (Whiteley *et al.*, 2001). Pf4 was induced when PAO1 cells encountered oxidative stress, and the primary oxidative stress response protein OxyR is involved in this process (Wei *et al.*, 2012; Hui *et al.*, 2014). It was reported that Pf4 superinfection in *P. aeruginosa* is regulated by BfmR which is part of a two‐component signal transduction pathway in response to membrane perturbing stress (Petrova *et al.*, 2011). A recent study showed that Pf5 production is activated a million‐fold by inactivation of the substrate binding protein DppA1 through an unknown mechanism linked to Pf5 nutrient sensing; i.e., when nutrients are low, Pf5 lyses the host (Lee *et al.*, 2018). Further studies are needed to explore whether these genetic factors function through the excisionase gene to regulate the Pf4 and Pf5 prophage induction or whether other host factors can directly activate Pf production under specific conditions.

**Figure 9:**
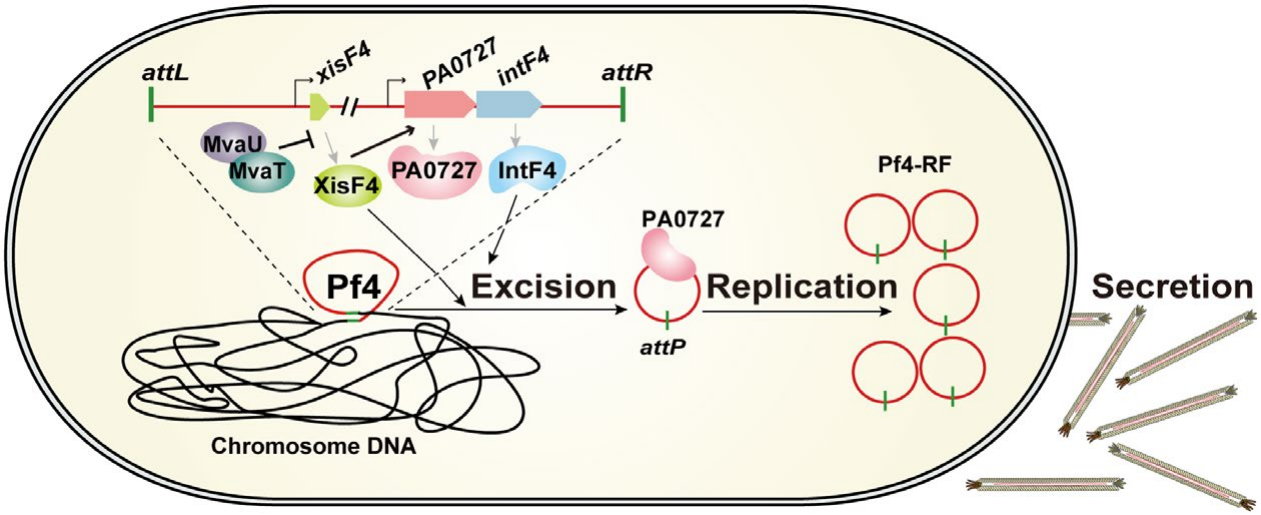
A proposed model of the lysis‐lysogeny switch of Pf4 in PAO1. The stable integration of Pf4 in the host genome is maintained by the host proteins MvaT/MvaU. MvaT and MvaU repress the expression of *xisF4 *by binding to the promoter of *xisF4*. When *mvaT *and/or *mvaU *are repressed or inactivated, expression of *xisF4* will be de‐repressed and induce Pf4 excision. Meanwhile, XisF4 can activate the expression of *PA0727* (replication initiator) and produce a large number of circular forms of Pf4. Finally, Pf4 will be assembled and secreted to the outside of the cell.

In PAO1, MvaT and MvaU can form homomeric or heteromeric complexes (Castang *et al.*, 2008). Under normal growing conditions, MvaT and MvaU repress the expression of *xisF4 *by binding to the *xisF4* promoter. As a result, Pf4 prophage stably resides in the host genome, and Pf4 replication is repressed. When *mvaT* and *mvaU* are repressed, *xisF4* will be derepressed. Subsequently, XisF4 will function as a recombination directionality factor and promote the excision of Pf4, along with the presence of integrase. Meanwhile, XisF4 will bind to the promoter region of PA0727, leading to the activation of PA0727. As a replication initiator protein, PA0727 will promote the replication of Pf4 (Fig. 9). In *E. coli*, H‐NS was stimulated by cold shock through the protein CspA (La Teana *et al.*, 1991). H‐NS is also repressed by an antisense RNA, called DsrA, along with an RNA‐binding protein called Hfq (Lease and Belfort, 2000; Brescia *et al.*, 2003). In *Salmonella enterica*, H‐NS is an essential component in thermoregulation that responds to the temperature change (Ono *et al.*, 2005). Recently, in *Shewanella oneidensis*, H‐NS was found to be responsible for the prophage excision during cold adaption (Zeng *et al.*, 2016). However, we found that decreasing the temperature from 37 to 15°C did not change the expression levels of *mvaT* and *mvaU *or the production of Pf4. In addition, Pf4 production was induced during biofilm formation (Whiteley *et al.*, 2001); one of the possible causes is that the *mvaT‐* and *mvaU*‐mediated repression of *xisF4* was blocked during biofilm formation. However, the stress factors and genes involved in the regulation of *mvaT* and *mvaU* in PAO1 have rarely been demonstrated. It is reasonable to speculate that the MvaT and MvaU might be counter‐silenced by other DNA‐binding proteins (Will *et al.*, 2015) or become inactivated in response to environmental stress and consequently increases phage production. Nevertheless, the underlying regulation of MvaT and MvaU in response to environmental stimuli would help understand the trade‐off between Pf filamentous phage and its bacterial host.

## Materials and methods

### Bacterial strains, plasmids and growth conditions

Bacterial strains and plasmids are listed in Table 1, and primers are listed in Table S1. *E. coli* and *P. aeruginosa* PAO1 strains were grown in Luria‐Bertani (LB) medium at 37°C unless specified otherwise. When necessary, the following antibiotics were added at the indicated concentrations: tetracycline (50 µg ml^–1^), gentamycin (30 µg ml^–1^), carbenicillin (100 µg ml^–1^), kanamycin (50 µg ml^–1^).

### Construction of deletion mutants

The gene deletion method used here in *P. aeruginosa* was previously reported (Hoang *et al.*, 1998). To delete Pf4, *xisF4*, *xisF5*, *intF5*, *pf4r*, *mvaT* and *mvaU*, upstream and downstream homologous sequences (0.7–1 kb) were amplified through PCR from PAO1 or PA14 genomic DNA. Gentamycin resistance gene was amplified through PCR from the plasmid pPS856. These three amplicons were then ligated into pEX18Ap to produce the deletion plasmids. In‐frame deletion mutants were obtained via homologous recombination using sucrose resistance selection. The Gm^R^ selectable marker was removed from the chromosome as described previously (Hoang *et al.*, 1998). For the construction of the double‐deletion mutant Δ*mvaT*Δ*mvaU*, the *mvaT* single‐deletion strain was used as the recipient for further deleting *mvaU*. The final obtained mutants were confirmed by sequencing.

### Construction of plasmids

For the construction of the expression plasmid pHERD20T, the full coding regions of *pf4r, pf5r, xisF4, xisF5, intF4 a*nd *intF5 *were amplified through PCR from PAO1 or PA14 genomic DNA, and the PCR products were purified, digested with restriction enzymes and ligated into the vector pHERD20T. For construction of promoter reporter strains, the putative promoter regions of *xisF4, pf4r, PA0720, PA0724 *and *PA0727 *were amplified by PCR. Each amplicon was ligated into mini‐CTX‐*lacZ* using the Vazyme ClonExpress II One Step Cloning Kit. The constructed plasmid was then transformed into PAO1 or ΔPf4 hosts and integrated into the chromosome at the *attB* site near the tRNA^Ser^ sequence (Becher and Schweizer, 2000). The tetracycline selection marker carried by the plasmid was removed from the chromosome as described previously (Hoang *et al.*, 1998).

### Plaque assay

Pf4 phages were collected from planktonic cultures of PAO1 strains. Two milliliters of culture was collected and centrifuged at 12000 rpm for 2 min, and the supernatant was filtered through a 0.22 μm filter (Millipore Millex GP) to obtain a pure Pf4 solution. Then, the supernatant was 10‐fold serially diluted using LB. For preparation of bacterial lawns, we used the top‐layer agar method as previously described (Eisenstark, 1967). Then, 1 ml of stationary culture OD_600_ ~ 4 (PAO1 or ΔPf4) was mixed with 4 ml of molten LB‐top agar (8 g^–l^ agar, 0.1% glucose, 5 mM CaCl_2_) at 50°C and poured over LB‐bottom plates (10 g^–l^ agar, 0.1% glucose, 5 mM CaCl_2_). Finally, 10 μl of the serially diluted Pf4 solution was applied to bacterial lawns, and the plaques were visualized after 8 h of incubation.

### Reporter activity assay

Specific β‐galactosidase activity (U mg^–1^) of strains harboring the *pf4r*, *xisF4*, *PA0720, PA0724 *and *PA0727* promoter reporter constructs was determined using the Miller assay (Miller, 1972). To determine the promoter activity of *pf4r*, *xisF4*, *PA0720, PA0724 *and *PA0727* under overexpression of *xisF4 *or *pf4r, *pHERD20T‐*xisF4* or pHERD20T‐*pf4r* was transformed into the strain carrying the reporter. Strains were grown overnight in LB supplemented with carbenicillin. The overnight cultures were diluted 1:100 in LB, and 10 mM arabinose was added from OD_600_ ~ 0.1. After induction for 3 h, cells were collected to determine *β*‐galactosidase activity. To determine the promoter activity of *xisF4* in PAO1, Δ*mvaT*, Δ*mvaU *and Δ*mvaT*Δ*mvaU*, strains were grown overnight in LB. The overnight cultures were diluted 1:100 in LB, and the cells were collected to determine *β*‐galactosidase activity when the OD_600_ was ~1.0.

### Protein purifications

Protein XisF4 and Pf4r were purified from *E.coli* BW25113 strain containing plasmid pHERD20T‐*xisF4* and pHERD20T‐*pf4r*, respectively. One liter of LB supplemented with carbenicillin was inoculated with 10 ml of overnight culture, and the bacteria were grown with shaking at 37°C. 10 mM arabinose was added from OD_600_ ~ 0.5 and all the cells were collected by centrifugation after induction for 6 h. Protein IntF4, XisF5 and IntF5 were extracted from *E. coli* BL21(DE3) strain containing plasmid pET28b‐*intF4*, pET28b‐*xisF5* and pET28b‐ *intF5*, respectively. One liter of LB supplemented with kanamycin was inoculated with 10 ml of overnight culture, and the bacteria were grown with shaking at 37°C. 0.5 mM IPTG was added from OD_600_ ~ 0.5 and all the cells were collected by centrifugation after induction for 6 h. The subsequent steps of extraction protein from the collected pellet were performed as previously described (Liu *et al.*, 2015).

### Electrophoretic mobility shift assay (EMSA)

Electrophoretic mobility shift assays were performed as previously described (Liu *et al.*, 2015). DNA fragment AttL (122 bp) and AttR (252 bp) flanking the left or right attachment site of Pf4 were amplified through PCR from the genomic DNA of PAO1 strain, using primer pair probe‐Pf4attL‐F/R or probe‐Pf4attR‐F/R (Table S1), respectively. DNA fragment AttB (184 bp) covering the attachment site of Pf4 were amplified from the genomic DNA of ΔPf4 strain, using primer pair probe‐Pf4attB‐F/R (Table S1). DNA fragment AttL of Pf5 (141 bp) flanking the left attachment site of Pf5 were amplified from the genomic DNA of PA14 wild‐type strain, using primer pair probe‐Pf5attL‐F/R (Table S1). DNA fragments of the intergenic region between *xisF4* and *pf4r* (232 bp), the promoter region of PA0720 (253 bp), PA0724 (282 bp) and PA0727 (229 bp) were amplified from PAO1 using the corresponding primer pair listed in Table S1. All the purified DNA fragments were labeled biotin by using the Biotin 3′ End DNA Labeling Kit (Termo scientific, Rockford, USA). DNA fragments (0.25 pmol) were mixed with the purified proteins and incubated at 25°C for 2 h. The binding reaction components were added following the protocol as described in the LightShiſt Chemiluminescent EMSA kit (Termo scientific, Rockford, USA). Then the binding reaction samples were run on a 6% DNA retardation gel at 100 V in 0.5 × TBE and were then transferred to nylon membranes. The membranes were visualized using the Chemiluminescence Nucleic Acid Detection Module Kit (Termo scientific, Rockford, USA).

### 5′‐RACE

Mapping of the 5′ transcriptional start site was performed using the SMARTer RACE 5′/3′ Kit (TAKARA) following the manufacturer’s recommendations. First, RNA was extracted using an RNA extraction kit (Promega, Madison, WI, USA). Second, polyA tails were added to RNA prior to first‐strand 3′‐cDNA synthesis using a Poly‐(A) Polymerase enzyme (Takara Bio Cat. No. 2180A). Third, the *pf4r*‐ and *xisF4*‐specific primers were designed following the protocol in the SMARTer RACE 5′/3′ Kit user manual. Finally, the inserts were sequenced using the M13R primer.

### Quantitative reverse‐transcription real‐time PCR (qRT‐PCR)

Strains were grown overnight and adjusted to an OD_600_ of 0.05 in LB. Cells were collected at OD_600 _~ 1.0 by centrifugation (12000 rpm for 1 min), and used for RNA extraction using an RNA extraction kit (Promega, Madison, WI, USA). The medium was supplemented with carbenicillin for strains carrying pHERD20T‐based plasmids, and 10 mM arabinose was added at OD_600_ ~ 0.8 for 30 min. Total RNA was extracted from exponential‐phase bacteria using The cDNA synthesis was conducted using reverse transcription (Promega, Madison, WI, USA). Total cDNA (50 ng) was used for qRT‐PCR using the Step One Real‐Time PCR System. The level of the *16S rRNA* gene transcript was used to normalize the gene expression data.

### Quantification of prophage genome excision and extrachromosomal phage copy number

The frequency of prophage excision and the extrachromosomal phage copy number under different conditions were quantified by quantitative PCR (qPCR). To determine the the frequency of Pf4 or Pf5 excision, the number of chromosomes that are devoid of Pf4 or Pf5 were quantified using primers (Pf4‐f/r or Pf5‐f/r) flanking the reconstituted bacterial attachment site (*attB*) (Fig. 2A), which only generate PCR products when the prophage is excised because of the size of the prophage (Fig. 1). The numbers of Pf4 or Pf5 RF molecules were quantified using primers (Pf4‐Cf/r or Pf5‐Cf/r) flanking the phage attachment site (*attP*) (Fig. 2A). The final value of frequency of excision and RF number were normalized by reference gene (*gyrB*), which is a single‐copy housekeeping gene indicating the number of total chromosomes. Strains were grown overnight and adjusted to an OD_600_ of 0.05 in LB. Cells were collected at OD_600 _~ 1.0 by centrifugation (12000 rpm for 1 min), and used for DNA extraction using DNA Isolation Kit (TIANGEN DP302). The medium was supplemented with carbenicillin for strains carrying pHERD20T‐based plasmids, and 10 mM arabinose was added from OD_600_ ~ 0.05. Total DNA (50–200 ng) was used as the template for the qPCR reaction using Maxima SYBR Green/ROX qPCR Master Mix (Thermo Fisher). The reaction was conducted using the Step One Real‐Time PCR System.

### Phylogenetic analysis

The distributions of XisF4 and XisF5 among a total of 1999 sequenced *Pseudomonas *strains were obtained by using the gene profile and alignment tool in the IMG/M system, version 4.6, with an *e*‐value of 0.01 and minimal amino acid identity of 60% (Mukherjee *et al.*, 2017). For phylogenetic analysis of XisF4 and XisF5, 266 homologous sequences were retrieved by BLASTp in the IMG/M system (*e*‐value of 0.01 and minimal amino acid identity of 35%), excluding XisF4 and XisF5 homologues whose neighboring genes are not from Pf prophages (Table S2) (Mukherjee *et al.*, 2017). A final set of 25 representative sequences (including only homologues with amino acid identity of 100%) was selected for construction of a phylogenetic tree in MEGA 5.0 using the neighbor‐joining (NJ) method with 1000 replications (Tamura *et al.*, 2011). For phylogenetic analysis of Pf prophages based on PA0724 (minor coat protein), 433 sequences were retrieved via BLASTp in the IMG/M system (*e*‐value of 0.01 and minimal amino acid identity of 35%), excluding PA0724 homologues whose neighboring genes are not from Pf prophages (Table S2) (Mukherjee *et al.*, 2017). The phylogenetic tree of PA0724 homologues was constructed with MAFFT using an averaged linkage (UPGMA) approach (Kuraku *et al.*, 2013). The corresponding neighboring gene, the XisF4 or XisF5 homologue, was displayed on the PA0724 tree based on the phylogenetic analysis of XisF4 and XisF5. A set of 397 homologues of IntF4 and of the neighboring gene, PA0724, were retrieved in the IMG/M system for phylogenetic analysis, and the phylogenetic tree of IntF4 was constructed using the same method as for PA0724 (Table S2). The neighboring genes of tRNA, *xisF4* or *xisF5* are displayed on the phylogenetic tree for IntF4. The placement of genes on trees was visualized and annotated using the iTOL interface (Letunic and Bork, 2016).

## Conflicts of interest

The authors declare no conflict of interests.
