# Supplementary material for: Excisionase in Pf filamentous prophage controls lysis‐lysogeny decision‐making in Pseudomonas aeruginosa
Source: Mol Microbiol. 2018 Dec 12;111(2):495–513. doi: 10.1111/mmi.14170 (PMC7379572; doi:10.1111/mmi.14170)
Supplement: Supplementary file 1 [file MMI-111-495-s001.docx]

**Supplementary File**

Excisionase in Pf Filamentous Prophage Controls Lytic-Lysogenic Decision-Making in *Pseudomonas aeruginosa*

Yangmei Li^1,2^, Xiaoxiao Liu^1^, Kaihao Tang^1^, Pengxia Wang^1^,Zhenshun Zeng^1^, Yunxue Guo^1^, Xiaoxue Wang^1,2*^

^1^Key Laboratory of Tropical Marine Bio-resources and Ecology, Guangdong Key Laboratory of Marine Materia Medica, RNAM Center for Marine Microbiology, South China Sea Institute of Oceanology, Chinese Academy of Sciences, Guangzhou 510301, PR China,

**^2^University of Chinese Academy of Sciences**, Beijing 100 049, China

*To whom correspondence should be addressed. Tel: +86 20 89267515; Fax: +86 20 89235490;

Email: xxwang@scsio.ac.cn

**Keywords:** excisionase, repressor *c*, *Pseudomonas aeruginosa*, PAO1

**Table. S1** Oligonucleotides used for plasmid construction, gene knockout, DNA sequencing, qRT-PCR, EMSA and 5’-RACE. F indicates forward primer and R indicates reverse primer.

| **Purpose/Name** | **Sequence(5'-3')** |
| --- | --- |
| **In-frame deletion** |  |
| pEX18Ap –F | AATCTTCTCTCATCCGCCAAAACA |
| pEX18Ap –R | CGCCCAATACGCAAACCGCCTCTC |
| Pf4-UpF-SacI | GCCCCCGAGCTCGTTATTGGTCGTGGTTGCTTCCC |
| Pf4-UpR- NheI | GCCCCCGCTAGCGATCCCAATGCAAAAGCCCC |
| Pf4-DnF-NheI | GCCCCCGCTAGCTGGAGCGGGCGAAGGGAATCGAACCCTCG |
| Pf4-DnR-HindIII | GCCCCCAAGCTTCAGCCTGGACGAGCACGAATACC |
| pPS856-Gm-F-NheI | GCCCCCGCTAGCCGAATTAGCTTCAAAAGCGCTCTGA |
| pPS856-Gm-R-NheI | GCCCCCGCTAGCCGAATTGGGGATCTTGAAGTTCCT |
| Pf4-RFF | AGCAGCGCGATGAAGCAAT |
| Pf4-RFR | TAGAGGCCATTTGTGACTGGA |
| Pf4-LF | TACGAGGCTGTTGAGGAGTTA |
| Pf4-LR | CCGTGCCGAGGTAGTGATGTC |
| Pf4-up | TGGTCGTGGTTGCTTCCCTGTT |
| Pf4-dn | CTTCCATATCCGCGAAGTGCTG |
| Pf4-RF-IF | AGGGCAACACAAACGTAAACA |
| Pf4-RF-IR | AGTCAGCCAGCAGGCGAGTC |
| *xisF4*1F | GCCAGTGCCAAGCTTGCATGCTGCCGGAATTGGCGCCTG |
| *xisF4* 1R | AAGATCCCCAATTCGGCACTGGGGCTAAGCTCTTCC |
| *xisF4* 2F | AGTGCCGAATTGGGGATCTTGAAGTTC |
| *xisF4* 2R | GCAGCTTCCCGAATTAGCTTCAAAAGCGC |
| *xisF4* 3F | AAGCTAATTCGGGAAGCTGCGCATGGTGAATAG |
| *xisF4* 3R | TATGACCATGATTACGAATTCGAGCGCGGGGCGGAGAAG |
| *xisF4* SF | TGGGTTTGGCGTAGGTAGTAG |
| *xisF4* SR | GTGAGAGGTGATTGAGGGGTA |
| *xisF4* LF | GTCGGTGGCGGGAGTAATG |
| *xisF4* LR | CAGCGGTGTTGGGTGAAAG |
| *pf4r*Up EcorI | CCGGAATTCGTGGGCTTCCCGCTGTCGTA |
| *pf4r*Up XbaIR | CTAGTCTAGACTATCGGCTGACGTGCTCATAT |
| *pf4r*Dn FXbaI | CTAGTCTAGAGGATAGCGATCACTAGAAAA |
| *pf4r*Dn RHindIII | CCCAAGCTTAACGGAACAGGTAAAGGTTG |
| *pf4r*SF | GCTCATTCCGTCACTATTCG |
| *pf4r*SR | TCATTTCTTGCCTTCCATCC |
| *pf4r*LF | TCAGGCAGAGCAGGAGCAG |
| *pf4r*LR | CCCTTTGGGCAGCGATTTA |
| *mvaT*-UpF-SacI | GCCCCCGAGCTCAAGCTGTGGTGGATGTTGATC |
| *mvaT*-UpR-XbaI | GCCCCCTCTAGAGTCAGGTACCTTGTCTGTGCT |
| *mvaT*-DnF-XbaI | GCCCCCTCTAGAACCAGTCAGTTCCACGAAGAACG |
| *mvaT*-DnR-BamHI | GCCCCCGGATCCACCGCAATGCTCTGATCGTCT |
| *mvaT* SF | CGACGGGAACGTGGAAATACGGG |
| *mvaT* SR | CGGAACTGCGTCAACGCTATTC |
| *mvaT* LF | CTGGCGAGCGGTGACCTTCTA |
| *mvaT* LR | TACGATTACCTCTACCAGTTGCG |
| *mvaU-*UpF-SacI | GCCCCCGAGCTCCCACCGACGCCTACCAACGCC |
| *mvaU*-UpR-XbaI | GCCCCCTCTAGATCGTTTCACTCCCGTTCTAAAAATA |
| *mvaU*-DnF-XbaI | GCCCCCTCTAGAGCCGGTTTTCCCGACGGCATCCTGC |
| *mvaU*-DnR-HindIII | GCCCCCAAGCTTGATTGGAGGGAGGAACGAGGG |
| *mvaU* SF | CCCACGGACATAAAGGTTTCA |
| *mvaU* SR | CGCCGACGACAAGGACTACG |
| *mvaU* LF | ATCGCCTCAGCGTCTATCCCT |
| *mvaU* LR | GGGAGGGTGCCTGATGTCGG |
| *xisF5*-upF | GCCAGTGCCAAGCTTGCATGCCAAACATGTTCATCGGTGTTTCTC |
| *xisF5*-upR | CTGATGGAAAGGGAGGAGTTCTG |
| *xisF5*-downF | AACTCCTCCCTTTCCATCAGCGCCGCGCGGAGGTCTT |
| *xisF5*-downR | TATGACCATGATTACGAATTCTAGCTCACTAAACGCTCCGGG |
| *xisF5*-SF | GTTTGGTGCAGGTAGTAGGGAC |
| *xisF5*-SR | TGCTCACCCATCGGTTGTAG |
| *xisF5*-LF | TCCACAAGGACTTGCGTATTG |
| *xisF5*-LR | GGATATGCCGAGTCTGAGAACA |
| *intF5*-upF | GCCAGTGCCAAGCTTGCATGCTCAGAGGTTCATCACGTCGAGG |
| *intF5*-upR | GCCGAGACGTCCTAGATTTTTCCTTT |
| *intF5*-down-F | AAAATCTAGGACGTCTCGGCCATCCTCAAGCTTGGTGA |
| *intF5*-down-R | TATGACCATGATTACGAATTCGTGGTCCAAGGGCCGCAT |
| *intF5*-SF | CGACTTGCGTTCCCATTCC |
| *intF5*-SR | GCGGTCTACGATCCCTTCTG |
| *intF5*-LF | TCCACAAGGACTTGCGTATTG |
| *intF5*-LR | GGATATGCCGAGTCTGAGAACA |
| **Cloning** |  |
| pHERD20T-F | ATCGCAACTCTCTACTGTTTCT |
| pHERD20T-R | TGCAAGGCGATTAAGTTGGGT |
| *xisF4*-F- ECORI | CCGGAATTCGATGGAACTGGAAGAGCTTAG |
| *xisF4*-R-Hindiii | CCCAAGCTTTCAATGGTGATGGTGATGATGGGCACTCCATTCCTGTTCCA |
| *pf4r*-F-EcorI | CCGGAATTCGATGAGCACGTCAGCCGATAG |
| *pf4r*-R-HindIII | CCCAAGCTTCTAGTGGTGGTGGTGGTGGTGTCCCGCGTTTTGATTGGACA |
| *intF4*-F-EcoRI | GGAATTCGATGTCGATCACCAAGCTCCCCG |
| *intF4*-R-XbaI | CGCTCTAGACTAGTGGTGGTGGTGGTGGTGACCTATGAGCGGGTTGAATCGA |
| pET28b*- intF4-*F | TTAAGAAGGAGATATACCATGTCGATCACCAAGCTCCC |
| pET28b*- intF4*-R | CGAGTGCGGCCGCAAGCTTTTAGTGGTGGTGGTGGTGGTGACCTATGAGCGGGTTGAATC |
| pET28b- *intF5*-F | TTTAAGAAGGAGATATACCATGGCGATCACCAAGCTTGA |
| pET28b-intF5-R | CGAGTGCGGCCGCAAGCTTAGTGGTGGTGGTGGTGGTGGGACGTCTCGACAAGTGTCG |
| pET28b- PA14_49010-F | TTTAAGAAGGAGATATACCATGGAAGTGGAAGAAATCAA |
| pET28b-PA14_49010-R | CGAGTGCGGCCGCAAGCTTAGTGGTGGTGGTGGTGGTGGAACTCCTCCCTTTCCATCA |
| *pf5r*-F-EcorI | CCGGAATTCGATGAGCATCACAGATAGAGC |
| *pf5r*-R-HindIII | CCCAAGCTTCTAGTGGTGGTGGTGGTGGTGTCCCGCGCTTTGACTGGTCA |
| *intF5*-F-ECORI | CCGGAATTCGATGGCGATCACCAAGCTTGA |
| *intF5*-R-XbaI | CTAGTCTAGATCAATGGTGATGGTGATGATGGGACGTCTCGACAAGTGTCG |
| *xisF5*-F-ECORIF | CCGGAATTCGATGGAAGTGGAAGAAATCAA |
| *xisF5*-R- HindiiiR | CCCAAGCTTTCAATGGTGATGGTGATGATGGAACTCCTCCCTTTCCATCA |
| miniCTXlacZ-F | CGGAAGAAGGTCAATCATAAAGG |
| miniCTXlacZ-R | TGAGCGAGGAAGCGGAAGAGC |
| CTXattB-up | GCTACCGCCGCCCCTACGA |
| CTXattB-dn | CCGATCAGCACCACGAACACG |
| pCTX-P*_pf4r_*-*lacZ* F | CCCGGGCGAATCGATAAGCTTCAACAGCCAGGTACGCAGCA |
| pCTX-P*_pf4r_*-*lacZ* R | ATCGCTAGTTAGTTAGGATCCATAGAAATTTATTCCGGGGA |
| pCTX-P*_xisF4_*-*lacZ* F | CCCGGGCGAATCGATAAGCTTCTTGCAATCCATAATGCGTAATTAG |
| pCTX-P*_xisF4_*-*lacZ* R | ATCGCTAGTTAGTTAGGATCCGCTCATTCCGTCACTATTCGTGG |
| pCTX-P*_PA0720_*-*lacZ*-F | GTCGACGGTATCGATAAGCTTACAGCCTCCGCTGGGGAGTG |
| pCTX-P*_PA0720_*-*lacZ*-R | ATCGCTAGTTAGTTAGGATCCGGTGTTTCTCCTTCAATTCG |
| pCTX-P*_PA0724_*-*lacZ-*F | GTCGACGGTATCGATAAGCTTCCGCAACCTGTGCATCGCCG |
| pCTX-P*_PA0724_*-*lacZ*-R | ATCGCTAGTTAGTTAGGATCCCAAAAAAGCCCCCTGCCGGA |
| pCTX-P*_PA0727_*-*lacZ-*F | GTCGACGGTATCGATAAGCTTCCCGGCGCGGTGGCCAGCAT |
| pCTX-P*_PA0727_*-*lacZ-*R | ATCGCTAGTTAGTTAGGATCCCTTTAACGATCCAAATTGGT |
| **Probe For EMSA** | |
| probe-*pf4r*-*xisF4*F | TGCGCTCCGCCCACCGTTCG |
| probe-*pf4r*-*xisF4* R | AATAGTCTTGCTCTATCGGC |
| probe-*PA0720*F | GGTGTTGCTCTGCGGGTTC |
| probe-*PA0720*R | GGTGTTTCTCCTTCAATTCG |
| probe-*PA0724*F | TTCGCCGGGGTGATCGACAC |
| probe-*PA0724*R | CGTAAGACCCCACACAAAAA |
| probe-*PA0727* F | TCGATTGGGAATTCCTCGC |
| probe-*PA0727* R | CTTTAACGATCCAAATTGGTA |
| probe-Pf4attL-F | TTAAAATAGGGTTTTGAAGCG |
| probe-Pf4attL-R | TCATGCAGGACCTGTCGAAA |
| probe-Pf5attL-F | TCGGCAAAGCCGAATCAGCG |
| probe-Pf5attL-R | CATTTGTCGCCGGAGTACCT |
| probe-Pf4attR-F | CCTCGACACTTCTTCGACACCT |
| probe-Pf4attR-R | AAACCCAGCGAAATCAACAAAA |
| probe-Pf4attB-F | CGCGGTAAAGCCTGAAAACA |
| probe-Pf4attB-R | GCCGCGCATCTGGTAAAA |
| **qRT-PCR** |  |
| 16SRNA-F | TGGTTCAGCAAGTTGGATGTG |
| 16SRNA-R | GTTTGCTCCCCACGCTTTC |
| *PA0715*-qF | GTTTTCGACTTCCCCTGCAA |
| *PA0715*-qR | CACACGACCCCAAACAAAGG |
| *xisF4*-qF | CCGCAACAGGATGTGGAG |
| *xisF4*-qR | GCACTCCATTCCTGTTCCAA |
| *PA0717*-qF | TGCGCTACCTCGTAGAGATTTG |
| *PA0717*-qR | AGCGGCGAGCTTCTTCTTC |
| *PA0720*-qF | GGCGTCGTCGAACTGTGGGT |
| *PA0720*-qR | GCTGGCGAACTTGCAGAGCA |
| *PA0724*-qF | TTTGCGAGCCTGATTCTGATG |
| *PA0724*-qR | GTCTGGTCACGGGCTTTCAT |
| *PA0727*-qF | GGCGTGCTGGATGATTTGG |
| *PA0727*-qR | GACAGTTGCAGGCCGTTGG |
| *Rorf0727*-qF | GCGGCTGACCTGGATTTATC |
| *Rorf0727*-qR | AGAGCGATGCTTGCCTTCTG |
| **qPCR** |  |
| PAO1*gyrB*-f | CAAGTACGAAGGCGGTCTGAAG |
| PAO1*gyrB*-r | GCAGAGCAGGTTCTCGTTGAA |
| Pf4-Cf | GGGCTTGGCAGGGTGATT |
| Pf4- Cr | TCATGCAGGACCTGTCGAAA |
| Pf4-f | CGCGGTAAAGCCTGAAAACA |
| Pf4-r | GCCGCGCATCTGGTAAAA |
| PA14*gyrB-*f | CACAGCATCCAGCGATACAAG |
| PA14*gyrB*-r | CGCGTTGCTTTCGATGAAGT |
| Pf5-Cf | TTATGACCAACACCCCAAACG |
| Pf5-Cr | CATTTGTCGCCGGAGTACCT |
| Pf5-f | GTGCTCTGGAATCCGGGTGT |
| Pf5-r | CGTTGAACAGGAGGAAATGGGT |
| **5’-RACE** |  |
| 5’RACE-*pf4r*-gsp | GATTACGCCAAGCTTGGGCGATGGAACCGCTTGCAATCC |
| 5’RACE-*xisF4*-gsp | GATTACGCCAAGCTTTGCGGTACACCTGCCGAGCGCGTT |
| M13-R | CAGGAAACAGCTATGACC |

**Figure. S1**

**
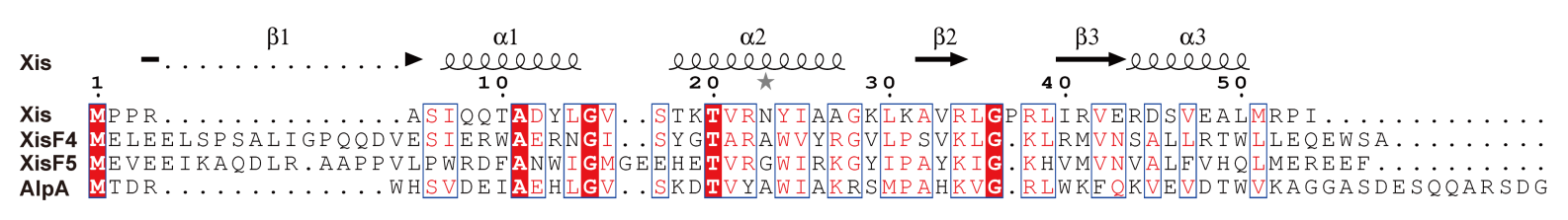
**

**Fig. S1. XisF4 and XisF5 alignment** Protein sequence alignment of Xis (mycobacteriophage Pukovnik ([Singh *et al.*, 2014](#_ENREF_3))), XisF4 (strain PAO1), XisF5 (strain PA14) and AlpA (strain *Pseudomonas mandelii*) was generated and analyzed using MAFFT([Kuraku *et al.*, 2013](#_ENREF_1)) and ESPript 3.x([Robert & Gouet, 2014](#_ENREF_2)). The secondary structure of Xis is displayed above the alignment. Identical residues are shown as white text on red background; similar residues are shown as red text.

**Figure. S2**

**
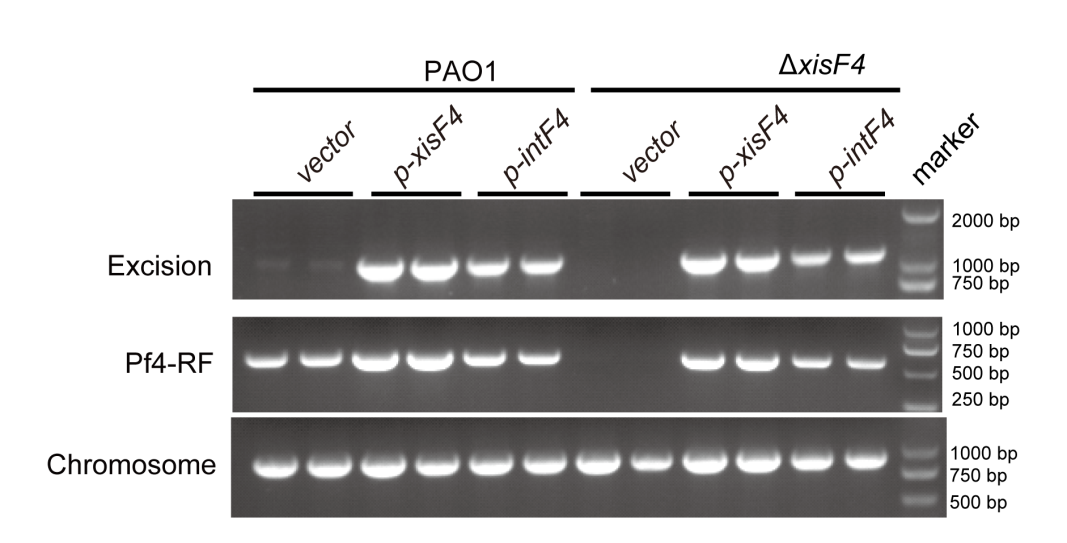
**

**Fig. S2. Pf4 excision and replication detected by PCR** The excision of Pf4 is detected by PCR using a forward primer (Pf4-up) flanking the left attachment site and a reverse primer (Pf4-dn) flanking the right attachment site of the prophage Pf4. The replication form of Pf4 (Pf4-RF) is detected by PCR using an inner forward primer near the left attachment site (Pf4-RF-IF) and an inner reverse primer near the right attachment site (Pf4-RF-IR) of the prophage Pf4. The lower panel which indicated the chromosome DNA is detected by PCR using a forward primer flanking the upstream of *mvaU* (mvaU-SF) and a reverse primer flanking the downstream of *mvaU* (mvaU-SR). The PCR templates are the PAO1 wild-type strain and *xisF4* deletion strain overexpressing *intF4* or *xisF4* via pHERD20T-based plasmids. The empty plasmid pHERD20T (p) was used as a negative control. Two independent cultures of each strain were used.

**.**

**Figure. S3**

**
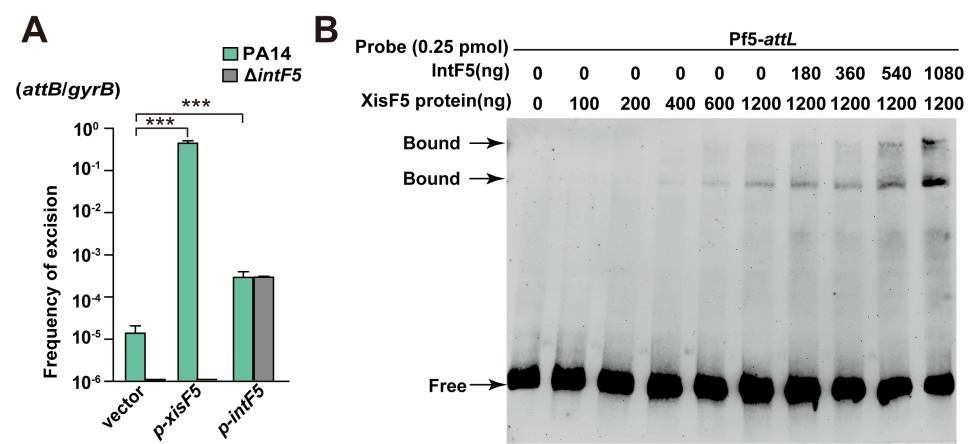
**

**Fig. S3. (A)** The frequency of Pf5 excision was quantified in strain PA14 and *intF5* deletion strain overexpressing *intF5* or *xisF5* via pHERD20T-based plasmids. The empty plasmid pHERD20T (p) was used as a negative control. Three independent cultures of each strain were used, and error bars indicate standard deviation. **(B)** EMSA showed that XisF5 and intF5 bound to *attL* of Pf5 in a concentration-dependent manner.

**Figure. S4**

**
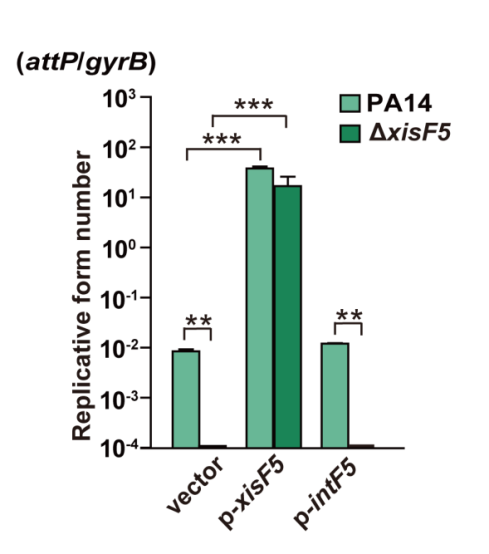
**

**Fig. S4.** The number of Pf5 RF molecules was quantified in the PA14 wild-type strain and *xisF5* deletion strain overexpressing *intF5* or *xisF5* via pHERD20T-based plasmids using the primer pair Pf4-Cf /Cr to quantify the number of *attP*. The relative RF (replicative form) number was normalized by the host reference gene *gyrB*. The empty plasmid pHERD20T (p) was used as a negative control. Three independent cultures of each strain were used, and error bars indicate standard deviation.

**Figure. S5**


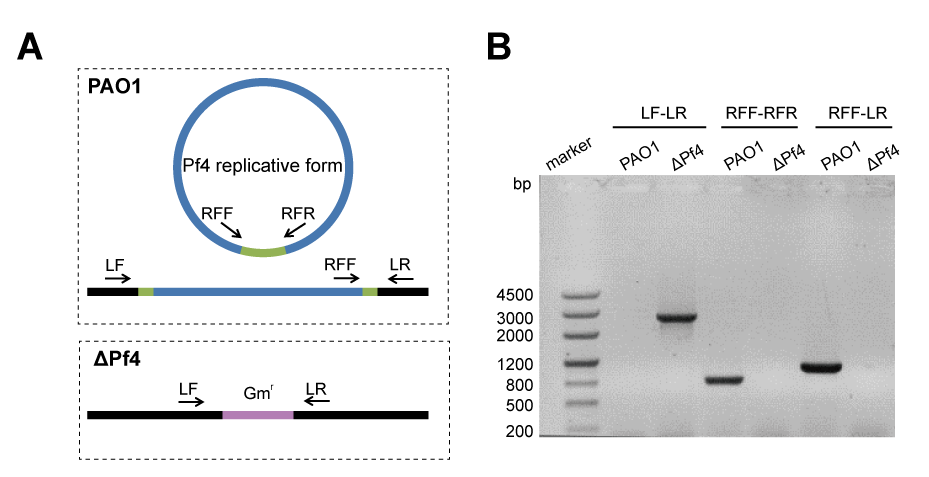


**Fig. S5. Deletion of Pf4 prophage from PAO1 was confirmed by PCR.** (**A**) Locations of the PCR primers are shown in the PAO1 strain (upper panel) and in the ΔPf4 strain (lower panel). (**B**) Deletion of the Pf4 prophage in the ΔPf4 strain was confirmed by the presence of the expected PCR product (2728 bp) using the primer pair LF-LR when Pf4 was removed from host genome. For the PAO1 strain, due to the size of the Pf4 prophage (12.4 kb), no PCR product was obtained using the same pair of primers (LF-LR). The absence of Pf4 prophage in the ΔPf4 strain was also verified by the absence of the extrachromosomal Pf4 circle by using primer pair RFF-RFR, which only amplifies the extrachromosomal Pf4 circle, and by the absence of PCR product when using the forward primer inside Pf4 (RFF) and the reverse primer outside Pf4 (LR). Wild-type PAO1 was used as a control.

**
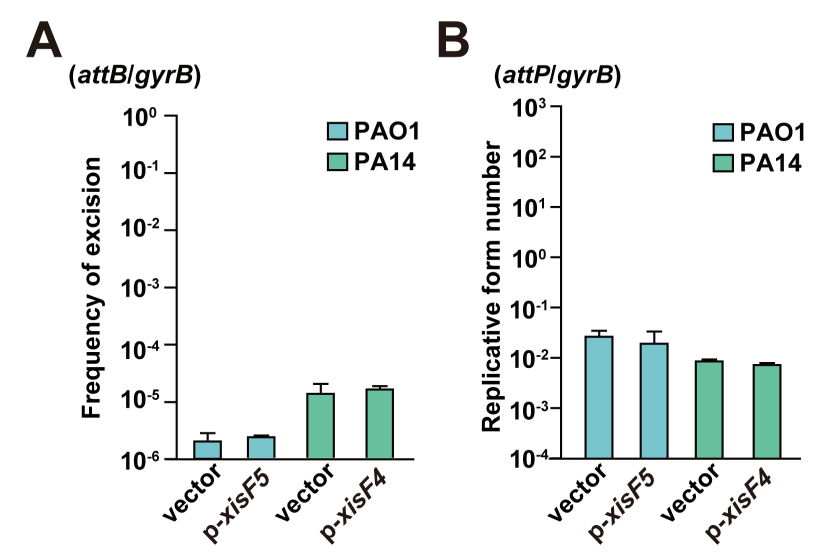
**

**Fig. S6. (A)** The frequency of Pf4 excision was quantified in strain PAO1 wild-type strain overexpressing *xisF5* via pHERD20T-based plasmids. The frequency of Pf5 excision was quantified in strain PA14 wild-type strain overexpressing *xisF4* via pHERD20T-based plasmids. The empty plasmid pHERD20T (p) was used as a negative control. **(B)**The numbers of Pf4 RF molecules were quantified in the PAO1 wild-type strain overexpressing *xisF5* via pHERD20T-based plasmids. The numbers of Pf5 RF molecules were quantified in the PA14 wild-type strain overexpressing *xisF4* via pHERD20T-based plasmids. The empty plasmid pHERD20T (p) was used as a negative control. Three independent cultures of each strain were used, and error bars indicate standard deviation.

**Figure. S7**


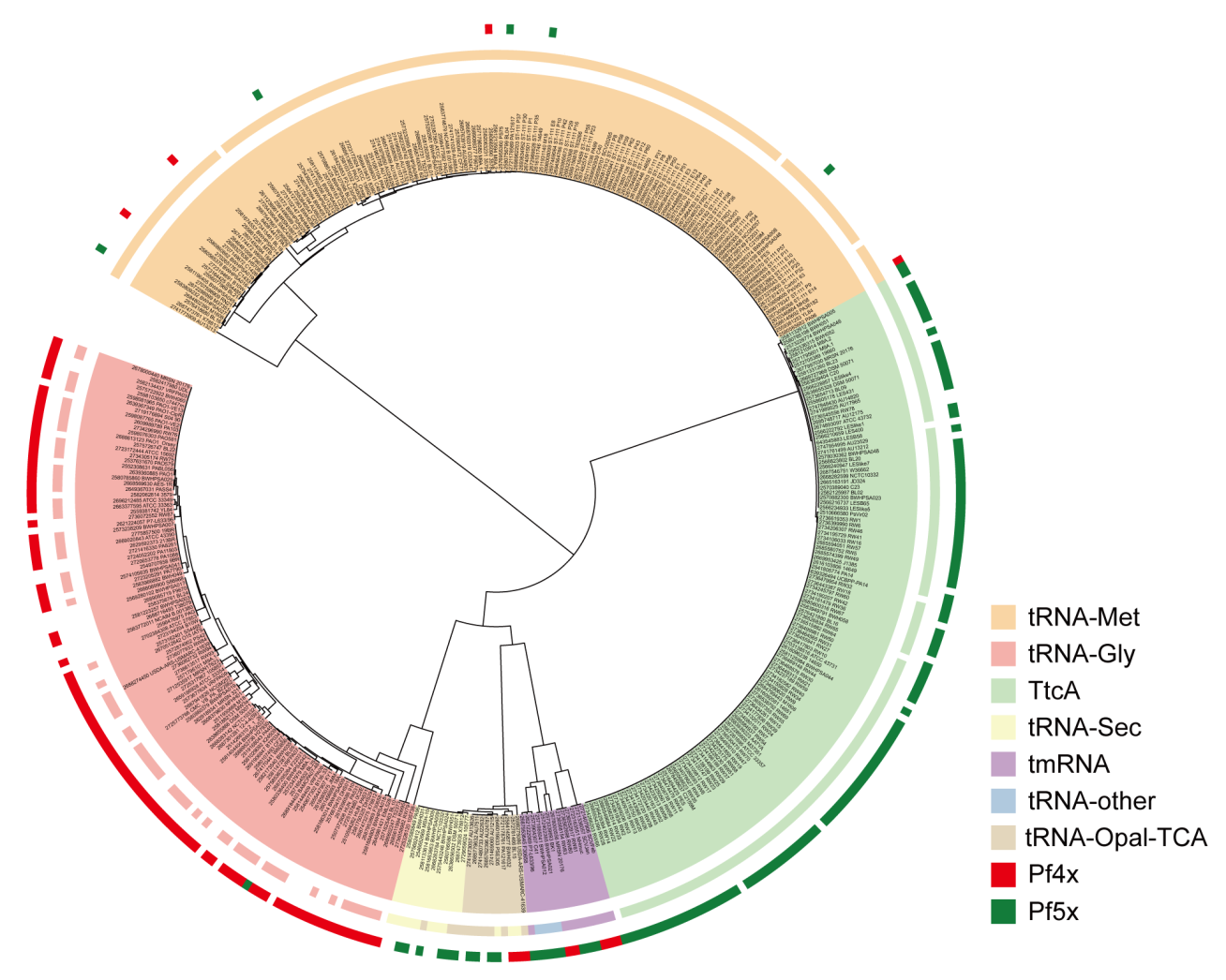


**Fig. S7. Phylogenetic analysis of PA0728** Phylogenetic tree of IntF4 (integrase) homologues indicated the attachment sites of Pf prophages. The first outer ring indicated the tRNA gene adjacent to the IntF4 homologue, which indicated the attachment site of the Pf prophage. The second outer ring represented the presence of XisF4 (red), XisF5 (green) in the specific Pf phage carrying the corresponding IntF4 homologue on each node.

**Reference**

Kuraku, S., C.M. Zmasek, O. Nishimura & K. Katoh, (2013) aLeaves facilitates on-demand exploration of metazoan gene family trees on MAFFT sequence alignment server with enhanced interactivity. *Nucleic. Acids. Res.* **41**: W22-28.

Robert, X. & P. Gouet, (2014) Deciphering key features in protein structures with the new ENDscript server. *Nucleic. Acids. Res.* **42**: W320-W324.

Singh, S., J.G. Plaks, N.J. Homa, C.G. Amrich, A. Heroux, G.F. Hatfull & A.P. VanDemark, (2014) The structure of Xis reveals the basis for filament formation and insight into DNA bending within a mycobacteriophage intasome. *J. Mol. Biol.* **426**: 412-422.
